# Evolutionary origin and gradual accumulation with plant evolution of the LACS family

**DOI:** 10.1186/s12870-024-05194-2

**Published:** 2024-05-31

**Authors:** Siyuan Zhou, Xiao Wu, Yubo Yuan, Xin Qiao, Zewen Wang, Mayan Wu, Kaijie Qi, Zhihua Xie, Hao Yin, Shaoling Zhang

**Affiliations:** State Key Laboratory of Crop Genetics & Germplasm Enhancement and Utilization, Sanya Institute of Nanjing Agricultural University, Nanjing Agricultural University, Nanjing, 210095 China

**Keywords:** LACS family, Phylogeny, Evolutionary origin

## Abstract

**Background:**

LACS (long-chain acyl-CoA synthetase) genes are widespread in organisms and have multiple functions in plants, especially in lipid metabolism. However, the origin and evolutionary dynamics of the LACS gene family remain largely unknown.

**Results:**

Here, we identified 1785 LACS genes in the genomes of 166 diverse plant species and identified the clades (I, II, III, IV, V, VI) of six clades for the LACS gene family of green plants through phylogenetic analysis. Based on the evolutionary history of plant lineages, we found differences in the origins of different clades, with Clade IV originating from chlorophytes and representing the origin of LACS genes in green plants. The structural characteristics of different clades indicate that clade IV is relatively independent, while the relationships between clades (I, II, III) and clades (V, VI) are closer. Dispersed duplication (DSD) and transposed duplication (TRD) are the main forces driving the evolution of plant LACS genes. Network clustering analysis further grouped all LACS genes into six main clusters, with genes within each cluster showing significant co-linearity. Ka/Ks results suggest that LACS family genes underwent purifying selection during evolution. We analyzed the phylogenetic relationships and characteristics of six clades of the LACS gene family to explain the origin, evolutionary history, and phylogenetic relationships of different clades and proposed a hypothetical evolutionary model for the LACS family of genes in plants.

**Conclusions:**

Our research provides genome-wide insights into the evolutionary history of the LACS gene family in green plants. These insights lay an important foundation for comprehensive functional characterization in future research.

**Supplementary Information:**

The online version contains supplementary material available at 10.1186/s12870-024-05194-2.

## Background

Lipids are a major class of important organic compounds in living organisms with various biological functions, including in membrane structural components, cell recognition, and energy storage [1]. Fatty acids (FAs) are the foundation of cellular lipid biosynthesis; they can serve as energy suppliers and storage materials in cells and can participate in cellular signaling. In addition, based on the diversity and specificity of FAs in living organisms, some individual FAs and their ratios can be utilized as biomarkers [2]. Therefore, it is highly important to conduct systematic research on key gene families involved in the synthesis, degradation, and oxidation pathways of FAs.

FAs can be divided into three categories based on the length of the carbon chain, namely, long-chain fatty acids (LCFAs), medium-chain fatty acids (MCFAs), and short-chain fatty acids (SCFAs) [3]. The carbon chain length of FAs in embryogenic plants usually ranges from 14 to 20 [4]. Acyl-CoA synthetase (ACS) is present on the outer membranes of the endoplasmic reticulum, mitochondria, and other structures and is classified into four categories based on the length of the substrate carbon chains of specific FAs: very long-chain acyl-CoA synthetase (VLACS, > C20), long-chain acyl-CoA synthetase (LACS, C14-C20), medium-chain acyl-CoA synthetase (MACS, C10-C12), and short-chain acyl-CoA synthetase (SACS, C6-C8). Free FAs are chemically inert [5, 6]. Both in the synthesis and degradation of lipids, free FAs must be activated by LACS to acyl-CoA in the presence of CoA, ATP, and Mg^2+^ and then participates in various biochemical reactions in cells, such as the synthesis of fats and β-oxidation of FAs, carbon chain extension of FAs, modification of proteins, and signal transduction of jasmonic acid [6]. LACS catalyzes the conversion of free FAs to acyl-CoA thioesters through a two-step reaction. First, free FAs react with ATP to produce adenosylated intermediates (acyl-AMPs) and release pyrophosphate. Subsequently, the carbonyl of the acyl-AMP is attacked by the thiol group of CoA to form acyl-CoA thioesters and release AMP [7, 8].

LACS, an important enzyme for FA metabolism in plants, is involved mainly in the synthesis or degradation of phospholipids, triglycerides (TAGs), cuticular wax, cutin, starch, and cork and the β-oxidation of FAs [6, 9]. Some of these molecules play a crucial role as barriers in resisting biotic and abiotic stresses, some as sources of energy storage, and some as agents of communication between pollen and the stigma [6, 10]. Ultimately, regulation of LACS activity influences multiple plant phenotypes, including organ fusion, male infertility, abnormal stratum corneum structure, delayed seed germination, and changes in seed oil content [6, 11, 12].

LACS is widely present in various organisms. A fatty ACS named *FadD*, which belongs to the AMP binding protein family, was identified in *Escherichia coli*. The function of *FadD* is to promote the transmembrane transport of exogenous FAs into the cell and activate them into fatty acyl-CoA [13]. In addition, Sorger and Daum [14] reported four LACS genes (*Faa1p* to *Faa4p*) in yeast, and mutants of these four genes exhibited defects in the activation of free FAs. In embryogenic plants, LACS participates in multiple metabolic pathways and is located in different organelles, such as the endoplasmic reticulum [15, 16], plastids [15, 17], chloroplasts [17, 18], and peroxisomes [19, 20]. Considering the widespread existence and functional importance of the LACS gene family in the plant kingdom, in-depth analysis of the characteristics and evolution of this family is highly important.

The LACS family has been identified in multiple plants, and there are significant differences in the number of LACS family members among different species, such as 9, 11, 11, 17, and 34 members in *Arabidopsis* [21], apple [22], corn [23], soybean [4], and *Brassica napus* [24], respectively. Due to the rapid development of high-throughput sequencing technology, many plant genomes have been published. Making it possible to identify the LACS family on a large scale in plants. Ayaz et al. [25] conducted whole-gene identification and analysis of the LACS gene family in 122 plant species, and the results showed that the evolutionary tree of LACS family members mainly consists of six clades and that the expression levels of LACS genes vary among both anatomical and developmental stages. However, the origin and evolutionary history of the LACS family are largely unknown.

To better understand the origin and evolutionary history of the LACS gene family, in this study, we comprehensively identified members of the family in 166 species that have been sequenced and are distributed at different evolved loci in plants. In addition, we also conducted phylogenetic analysis, clade structure characterization, synteny network analysis, gene duplication event analysis, and Ka/Ks analysis and combined the results with data from species such as fungi to support relevant conclusions. Finally, we identified the origins and evolutionary relationships of different clades of plant LACS genes and present a hypothetical model of their evolutionary history. This study deepens the understanding of LACS in plants, and phylogenetic insights help further reveal the molecular and biological functions of various LACS proteins.

## Methods

### Collection and processing of genomic data files

The genomes of 222 species used in this study were collected from publicly available databases, such as NCBI, Phytozome, Ensembl, GigaDB, GDR, and Plant GARDEN [26–31]. The longest transcript of a single gene was extracted from the genomic data of each species.

### Identification of LACS gene family members

Nine *Arabidopsis* LACS protein sequences downloaded from TAIR (https://www.arabidopsis.org) [32] were used as queries to identify candidate LACS genes in the collected plant genomes via BLASTP (v2.13.0 +). The LACS domain seed file (PF00501) was downloaded from Pfam (http://pfam.xfam.org/) [33] to construct the Hidden Markov Model (HMM), and HMMER (v3.2.1) was used to identify the collected genomes. The E-value in both BLAST (v2.13.0 +) and HMMER (v3.2.1) software was set to less than e-20, and the overlapping genes from the two sets of results were considered candidate LACS family genes. Furthermore, the protein sequences of these genes were submitted to the CDD (https://www.ncbi.nlm.nih.gov/cdd) and SMART (http://smart.embl-heidelberg.de) databases [34] for domain detection (AMP binding domain), and if it was present, the gene was designated a member of the LACS family.

### Multiple sequence alignment and phylogenetic analysis

Multiple sequence alignment of all LACS protein sequences was performed by MAFFT (v7.520) (https://mafft.cbrc.jp/alignment/software/) with the parameter ‘E-INS-I’ [35]. After alignment, the sequences were trimmed using TrimAl (http://trimal.cgenomics.org), and the parameters gappyout or automated1 were selected [36].

The phylogenetic tree was constructed using IQ-TREE 2 (v2.2.0) (http://www.iqtree.org) with the maximum-likelihood method [37]. The optimal model parameter MFP for automatic detection was selected, and the bootstrap value was set to 1000. The species classification tree was obtained from NCBI Taxonomy Common Tree (https://www.ncbi.nlm.nih.gov/Taxonomy/CommonTree/wwwcmt.cgi). All phylogenetic trees were visualized through iTOL (https://itol.embl.de) Visualize [38].

### Identification of conserved domains and conserved motifs of LACS gene family members

The protein sequences of the conserved domains of the LACS family genes were subjected to multiple sequence alignment through MAFFT (v7.520) (https://mafft.cbrc.jp/alignment/software/). Then, the results were submitted to WebLogo3 (https://weblogo.threeplusone.com) for visualization [39, 40]. The protein sequences of the LACS gene family members were submitted to MEME (https://meme-suite.org/meme/tools/meme) for conservative motif identification. The parameters were set to a maximum of 20 motifs and a motif length range of 3–300 [41].

### Identification of duplicate genes

We used the method of Qiao et al. [42] to identify duplicate gene pairs in members of the LACS gene family through the DupGen_finder pipeline. Whole-genome protein sequence self-alignment was used to obtain a list of homologous gene pairs for each species through BLASTP (v2.13.0 +). The E-value was set to less than 10–5. Then, the top five results with the best matches were taken, and the output format was set to m8. The genomes of each species were converted into BED format and then examined to detect five types of duplication events, namely, whole-genome duplication (WGD), tandem duplication (TD), proximal duplication (PD), transposed duplication (TRD), and dispersed duplication (DSD).

### Cluster analysis of LACS gene family member networks

The SynNet pipeline was used for network clustering analysis [43]. First, pairwise alignment of protein sequences of the whole genome within and between species was performed for 166 species using the software Diamond (v0.9.14.115) [44]. Second, a collinearity block file containing 166 whole-genome protein sequences of the species was obtained by using the SynNet-Build algorithm. Information about members of the LACS gene family was extracted from the collinearity block file and integrated to obtain the collinear network relationships of the LACS family. Third, the network clustering relationships of LACS gene family members was visualized in Gephi (v0.10.1) using the Clique percolation method with a K value of 4 [45].

### Collinearity analysis

Sequence alignment of whole-genome proteins was performed through Diamond software, with an E-value of 10–5. Then, MCScanX software was used to detect collinearity between pairs of species and within species [46], which were visualized through iTOL (https://itol.embl.de) [38].

### Calculation of Ka and Ks replacement rates and Ka/Ks ratios for duplicated genes

ParaAT2.0 was used to integrate the process before calculating the Ka/Ks values [47]. The protein sequence alignment tool used for gene pairs was MAFFT (v7.520) (https://mafft.cbrc.jp/alignment/software/). Kaks_calculator2.0 was used to calculate Ka and Ks values, with the YN model [48].

## Results

### Identification of LACS family genes in plants

**Fig. 1:**
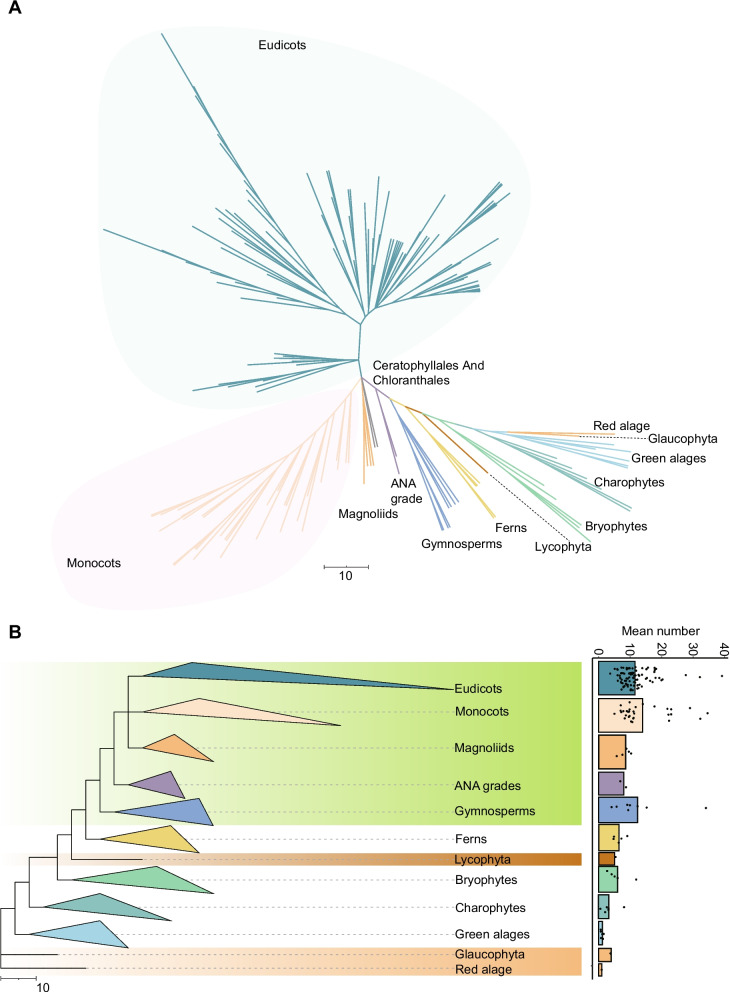
The phylogenetic relationships and distribution of LACS gene family members among 166 species. **A** Classification and phylogenetic tree of 166 species; **B** Diagram of the developmental relationship between major plant lineages and the distribution of the number of LACS gene family members in each clade

To comprehensively identify LACS genes in plants, we collected genomes from 166 species covering different plant lineages (Fig. 1A, Supplementary Table S1). The protein sequence encoded by each of the nine LACS family genes in *Arabidopsis* was used as a query in the BLASTP search to identify putative homologs in each species. An HMM was constructed from the AMP-binding domain seed file (PF00501) and used to determine whether the genes previously identified by the BLASTP search encoded the conserved domains. Ultimately, we identified 1785 members of the LACS gene family in 166 species (Supplementary Table S2). LACS genes were detected in all species investigated, from red algae, green algae, ferns, and gymnosperms to flowering plants, indicating that an ancient origin of the LACS gene family. The number of LACS genes varied dramatically among the 166 species, ranging from 1 (*Cyanidioschyzon merolae*, *Micromonas pusilla*, *Chlorella variabilis NC64A*, *Ulva mutabilis*, *Volvox carteri*, *Chlamydomonas reinhardtii*, *Chlorokybus atmophyticus-CCAC 0220*) to 39 (*Medicago sativa*) (Fig. 1B, Supplementary Table S2). With plant evolution, the overall number of LACS family genes gradually increased, with an average of 1.167 in green algae, 3.333 in charophytes, 6 in bryophytes, 6.4 in ferns, and 11.957 in seed plants (Fig. 1B, Supplementary Table S2).

### Phylogenetic analysis of LACS family genes in plants

**Fig. 2:**
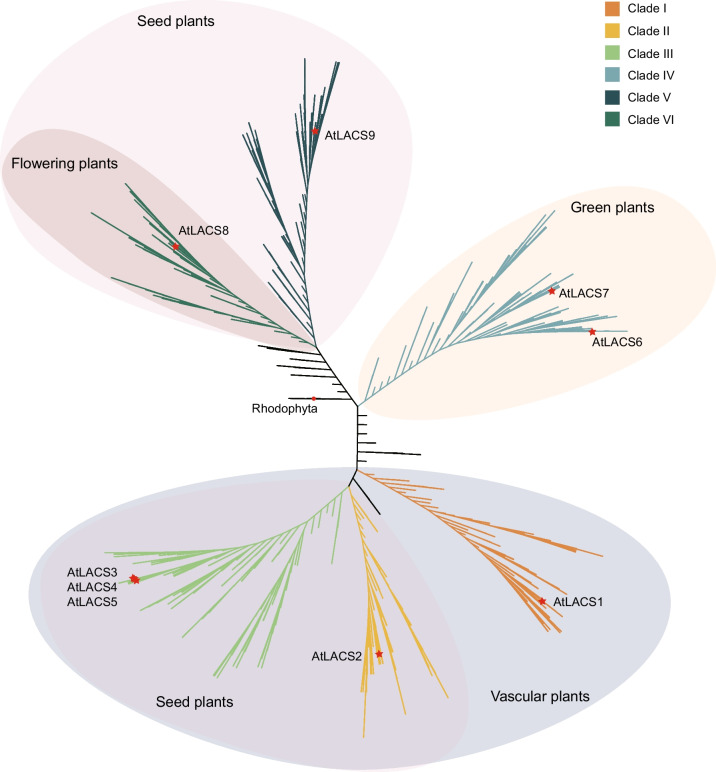
A phylogenetic tree of members of the LACS gene family. A phylogenetic tree of LACS gene family members identified from 166 species clustered into six main clades (I, II, III, IV, V, VI), with different background colors used to distinguish plant lineages belonging to different clades. The LACS gene of red algae (*Cyanidioschyzon merolae*) is marked with a red circle, and the LACS genes of *Arabidopsis thaliana* are marked with a red star

To explore the phylogenetic relationships of the LACS genes in plants, the 1785 LACS proteins were subjected to multiple sequence alignment using MAFFT, followed by trimming using TrimAl software, and the phylogenetic tree was constructed using IQ-TREE software with the maximum-likelihood method (Fig. 2). The members of the LACS gene family were mainly divided into six clades (I, II, III, IV, V, VI). The six clades contained 243 (Clade I), 213 (Clade II), 414 (Clade III), 364 (Clade IV), 288 (Clade V) and 197 (Clade VI) LACS genes. Notably, members of Clades IV, I and VI were detected in all the investigated green plant, vascular plant, and flowering plant species, respectively, while members of Clades II, III, and V were detected in all the investigated seed plant species (Fig. 2). The other 66 LACS genes were not classified into these six clades and are marked with black lines in the phylogenetic tree shown in Fig. 2; these genes are referred to as “Other Class Genes”. Among them, one belonged to rhodophytes, four to glaucophytes, one to chlorophytes, 13 to charophytes, 21 to bryophytes, 21 to ferns, three to lycophytes, and two to eudicots.

### The origin and evolution of different clades of plant LACS genes

**Fig. 3:**
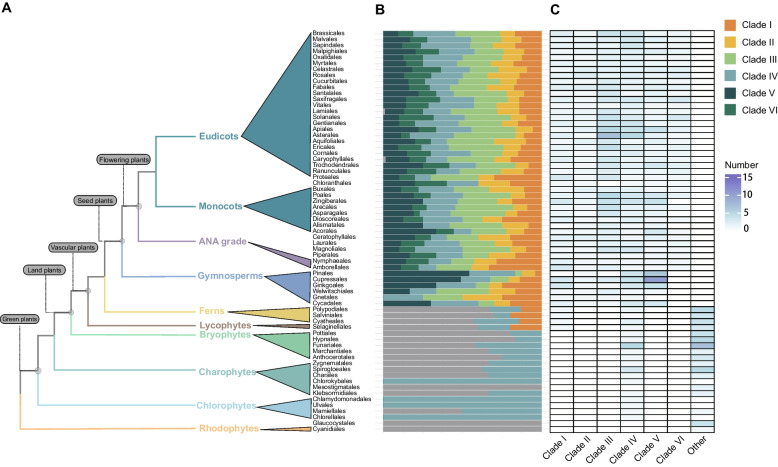
The distribution of LACS genes on different clades. **A** The developmental relationship pattern of plant lineages mainly focuses on the relationships between families. **B** Stacked plot of the percentage of LACS genes on different clades of each family, with each clade distinguished by a different color and each bar corresponding to the family in (**A**). **C** A heatmap of the number of LACS genes on different clades of each family, with colors ranging from white to blue representing the number from least to greatest and horizontally corresponding to each row in (**A**) and (**B**)

Considering that different clades of the LACS family exist in different plant lineages (Supplementary Table S3) and the evolutionary process of plant lineages (Fig. 3A), we analyzed the order of evolution of different clades in the phylogenetic tree. Clades IV, I, and VI appeared earliest in chlorophytes, lycophytes, and ANA grades, respectively, while Clades II, III, and V appeared earliest in gymnosperms, indicating that the order of appearance of the six clades was IV, I, (II, III, V), and VI (Fig. 3B, C). The LACS genes that were not classified among these six clades were the last to appear in ferns (Fig. 3). We further constructed individual phylogenetic trees for the clades by performing multiple sequence alignments of the genes from the six clades using the same method. The plant lineage that appeared earliest on each clade was consistent with the phylogenetic tree results constructed for all genes (Supplementary Figs. S1-S6).

Clade IV was the first clade of the LACS gene family to emerge during plant evolution. To determine whether Clade IV first appeared in chlorophytes or this was a spurious result due to the small number of investigated species, we identified 26 members of the LACS gene family in the recollected genomes of three rhodophytes, seven chlorophytes, and three fungal species using the same method. Then, we constructed a phylogenetic tree by combining these 26 LACS genes with previously identified LACS genes from rhodophyta, glaucophyta, and chlorophyta, as well as nine LACS genes from the model plant *Arabidopsis* (Supplementary Fig. S7, Table S4). The results showed that only two *AtLACSs* (*AtLACS6* and *AtLACS7*) of Clade IV were on the same clade as 12 of 17 LACS genes of chlorophytes, *AtLACS8* of Clade VI and *AtLACS9* of Clade V were on the same clade as 50 of 88 LACS genes of rhodophytes, chlorophytes, and fungi, and the other five *AtLACSs* were clustered together. Therefore, we believe that Clade IV first appeared in chlorophytes.

**Fig. 4:**
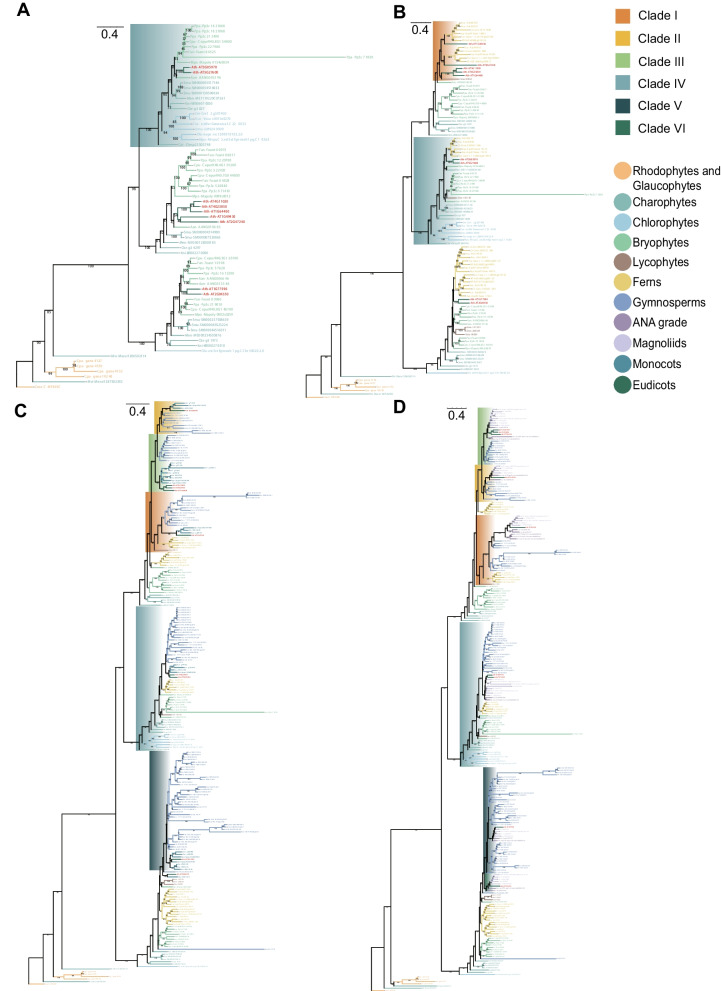
Analysis of the phylogenetic relationship of each clade origin. Different background colors represent different clades, and the different clade colors and fonts represent different plant groups. **A** The origin of Clade IV; **B** the origin of Clade I; **C**. the origin of Clade (II, III, V); **D** the origin of Clade VI. AtLACSs are represented in red font. The species selected for each phylogenetic tree (**A**-**D**) can be found in Supplementary Tables S5-S8

To further elucidate the origins of the different clades, we selected plant lineage-related species dating from rhodophyta to the first appearance of Clade IV, Clade I, Clade (II, III, V), and Clade VI and constructed separate evolutionary trees (Fig. 4, Supplementary Tables S5-8). Due to the limited number of genes at the first appearance of Clades II, III, and VI among the plant taxa, the clades of the phylogenetic tree could not be separated. We selected species from the plant lineages arising after the clade occurred to increase the sample size and constructed a phylogenetic tree by combining the LACS family genes identified by comparing these species with *Arabidopsis*. First, we constructed a phylogenetic tree based on the LACS genes identified in the rhodophyte, glaucophyte, chlorophyte, charophyte, and bryophyte species investigated (Fig. 4A, Supplementary Table S5). The results showed that Clade IV, containing *AtLACS6* and *AtLACS7*, separated from the other LACS genes as an independent clade, which further explains why Clade IV differentiated before the emergence of the green plants. Second, we constructed a phylogenetic tree based on the LACS genes identified in the investigated species of rhodophytes, glaucophytes, chlorophytes, charophytes, bryophytes, ferns, and lycophytes (Fig. 4B, Supplementary Table S6). Using *Mvi-Mesvi1206S02114* in charophytes as the node of the outgroup, two clades were differentiated, with one clade containing Clade IV, Clade I, 16 Other Class Genes and *AtLACS1-7* and the other clade including only 37 Other Class Genes and two *AtLACSs* (*AtLACS8* and *AtLACS9*) (Fig. 4B, Supplementary Table S6). Clade IV and two *AtLACSs* (*AtLACS6* and *AtLACS7*) formed a single clade, while Clade I, 16 Other Class Genes and *AtLACS1-5* cluster together on the same clade, and the corresponding species on this clade did not include chlorophytes. *AtLACS1-5* was not separated in the phylogenetic tree, possibly because *Arabidopsis* is a dicotyledonous plant, and this phylogenetic tree did not contain dicotyledonous plant species. The significant differences between species led to the clustering of five closely related genes (*AtLACS1-5*). We speculate that the evolutionary process of Clade I was independent of that of the chlorophytes, and the phylogenetic tree in Fig. 2 shows that the 16 LACS genes derived from charophytes and bryophytes are the outgroups of Clade I. These results suggest that the ancestors of Clade I existed earliest in charophytes. Third, we constructed a phylogenetic tree based on the LACS genes identified in the rhodophyte, glaucophyte, chlorophyte, charophyte, bryophyte, fern, lycophyte, gymnosperm and three monocot species investigated (Fig. 4C, Supplementary Table S7). Using *Mvi-Mesvi1206S02114* in charophytes as the node of the outgroup, two clades were differentiated, with one clade containing Clade V and 39 Other Class Genes and the other clade including only Clade (I, II, III, IV) and 22 Other Class Genes (Fig. 4C, Supplementary Table S7). The clade on which Clade V was located contained *AtLACS9*, and *AtLACS8* was closely related to this clade. The LACS genes in ferns constitute this subset of genes and were used as the outgroup. The clades of Clade (II, III) and Clade I share the LACS gene in ferns as the outgroup (Fig. 4C, Supplementary Table S7), indicating that the differentiation of Clade (II, III, V) may have occurred in a common ancestor with ferns. Fourth, we constructed a phylogenetic tree based on the LACS genes identified in the rhodophyte, glaucophyte, chlorophyte, charophyte, bryophyte, fern, lycophyte, gymnosperm, ANA grade and magnoliales species investigated (Fig. 4D, Supplementary Table S8). The results showed that the clade on which Clade V was located and that contained *AtLACS8* was separated from Clade VI and significantly separated from the other clades. The clade was more closely related to the LACS genes from ferns and lycophytes in the Other Class Genes group (Fig. 4D, Supplementary Table S8). This result suggested that Clades V and VI share a common ancestor with ferns.

In addition, we found that Other Class Genes were present mainly in plant lineages living before the gymnosperm evolutionary site arose, with only one Other Class Gene (*Lamiales-SIN_1001133* and *Caryophyllales-Dca16844.1*) found in *Dianthus caryophyllus* and *Sesamum indicum*, two eudicot species, respectively. We expanded the number of species in the eudicot orders to reidentify LACS genes (Supplementary Table S9). No additional Other Class Genes were found, and the phylogenetic tree showed that the two Other Class Genes (*Lamiales-SIN_1001133* and *Caryophyllales-Dca16844.1*) were not significantly clustered with the other LACS genes (Supplementary Fig. S8). We speculate that because the selected genomic data were collected in earlier years, when sequencing technology and assembly quality were relatively poor, errors occurred in these two gene sequences [49, 50]. A phylogenetic tree (Supplementary Table S10) was constructed for analyzing Other Class Genes. The results showed that the Clade (I, II, III, IV, V) all had an outgroup composed of Other Class Genes from bryophytes, ferns, and lycophytes (Supplementary Fig. S9), indicating a close correlation between Other Class Genes and Clade (I, II, III, IV, V) genes, and these clades shared a common ancestor with their corresponding outgroup from the genes of Other Class Genes.

### Gene structural characteristics of different clades

**Fig. 5:**
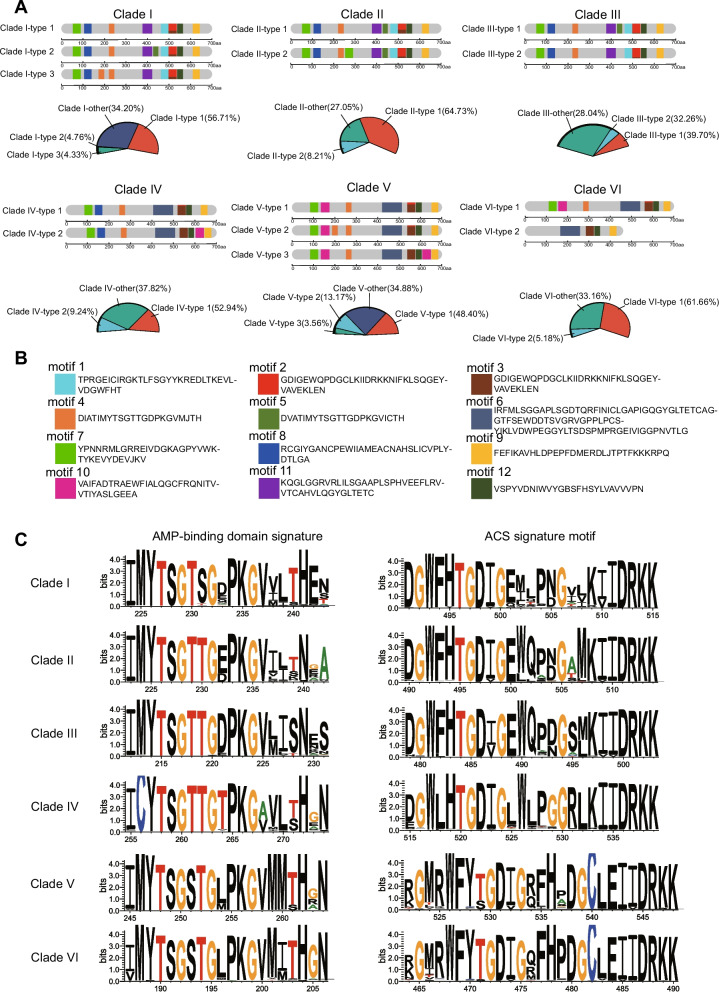
Analysis of conserved motifs and conserved domains of the six clades. **A** A pattern diagram of conserved motifs on six clades was obtained and statistically analyzed using the MEME tool, and similar genes with 10 or more conserved motif categories and positions were counted and classified into one type. A pie chart displaying the proportion of each type; **B** Each motif represents the base sequence; **C** Differential pattern diagram of six clades for the two conserved domains (AMP-binding domain signature and ACS signature motif)

The threshold for the maximum number of conserved motifs was set to 20 in MEME software, and a total of 12 conserved motifs of the LACS gene family proteins were detected. Specifically, two pairs of conserved motifs (motif 2 and motif 3, motif 4 and motif 5) were highly similar, and Clade (I, II, III) had more conserved motifs than Clade (IV, V, VI), indicating that Clade (I, II, III) is more conserved than Clade (IV, V, VI). To understand the types of conserved motif patterns on different clades, similar genes with 10 or more conserved motif categories and positions were counted and classified into one type (Fig. 5A, B, Supplementary Fig. S10, Table S11). Clades I and V had three types, and the other four clades had two types. The representation of Type 1 on each clade was the highest, reaching 39.70%-64.73%, while the other types had one to three motifs duplicated or missing compared to those in Type 1. The structures of the seven types in Clade (I, II, III) were generally similar, as were the structures of the three types in Clade V and Type 1 in Clade VI. Clade (I, II, III) had a greater proportion of motif 2 than Clade (V, VI), reaching 59.70–97.50%, while Clade (V, VI) had a higher proportion of motif 3, reaching 91.18–94.96%. Compared to Clade (V, VI), Clade (I, II, III) had a unique motif 1, motif 8, and motif 11, while Clade (V, VI) had a unique motif 6 and motif 10. In addition, the front-ends of three motifs in Clade IV were consistent with those in Clade (I, II, III), and the back-ends of four to five motifs in Clade IV were consistent with those in Clade (V, VI). Multiple alignment results of the protein sequences of LACS genes from Clade (I-VI) were analyzed using MAFFT and visualized using WebLogo3 (Fig. 5C). The results revealed differences in the two conserved domains (AMP-binding domain signature and ACS signature motif) of the LACS family genes across the six clades. For these two conserved domains, the similarity between Clade I, Clade II, and Clade III was greater, and the similarity between Clade V and Clade VI was greater, while Clade IV was significantly different from the other five clades, indicating that Clades I, II, and III had a closer genetic relationship, Clades V and VI had a closer genetic relationship, and Clade IV was most distantly related to the other five clades. This finding is also consistent with the results of the phylogenetic tree.

### Analysis of duplicate genes, synteny relationships, and Ka/Ks values on different clades

**Fig. 6:**
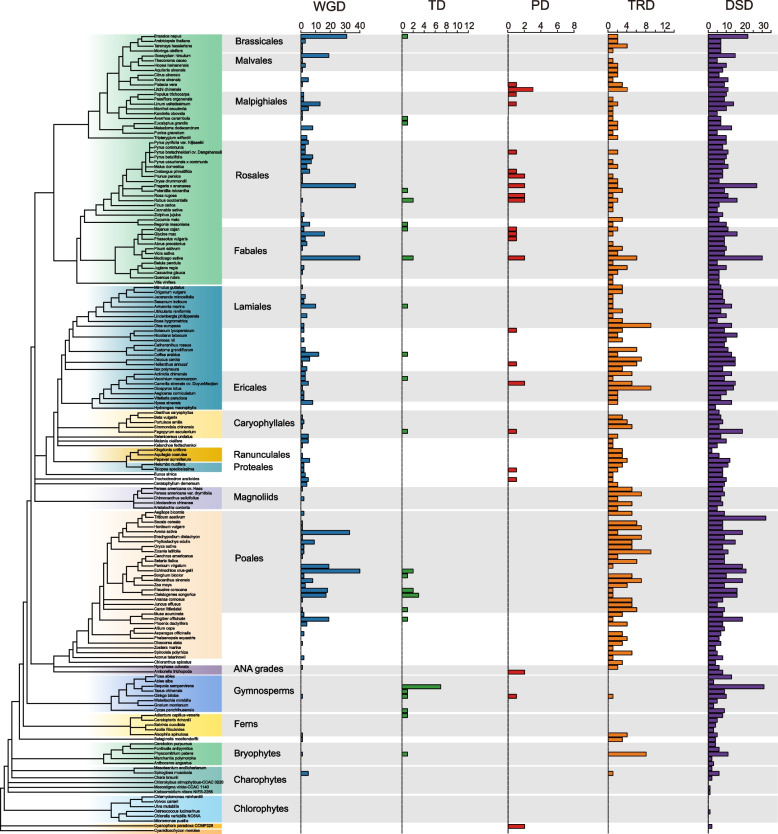
The number of pairs of LACS gene family members from five duplication events in 166 species genomes. The classification tree of 166 species was obtained from the NCBI Taxonomy Common Tree (https://www.ncbi.nlm.nih.gov/Taxonomy/CommonTree/wwwcmt.cgi). The bar chart shows the number of LACS gene pairs for each species in five duplication events: whole-genome duplication (WGD), tandem duplication (TD), proximal duplication (PD), transposed duplication (TRD), and dispersed duplication (DSD)

To further elucidate the evolutionary relationships of the LACS gene family, we first identified duplication events and duplicate gene pairs across the whole genomes of 166 species using the process of DupGen_finder (Fig. 6, Supplementary Fig. S11, Table S12). A total of five gene duplication events were identified, including WGD, TD, PD, TRD, and DSD. The number of duplicated gene pairs in the LACS gene family varied greatly among the different species, ranging from none identified in 7 algae and 80 pairs identified in *Medicago sativa*. Five types of gene duplication contributed to the expansion of the LACS gene family. The proportion of duplication events attributed to DSD, TRD, and WGD was greater than that attributed to TD and PD, with DSD and TRD interpreted as the main forces driving the expansion of the LACS gene family.

**Fig. 7:**
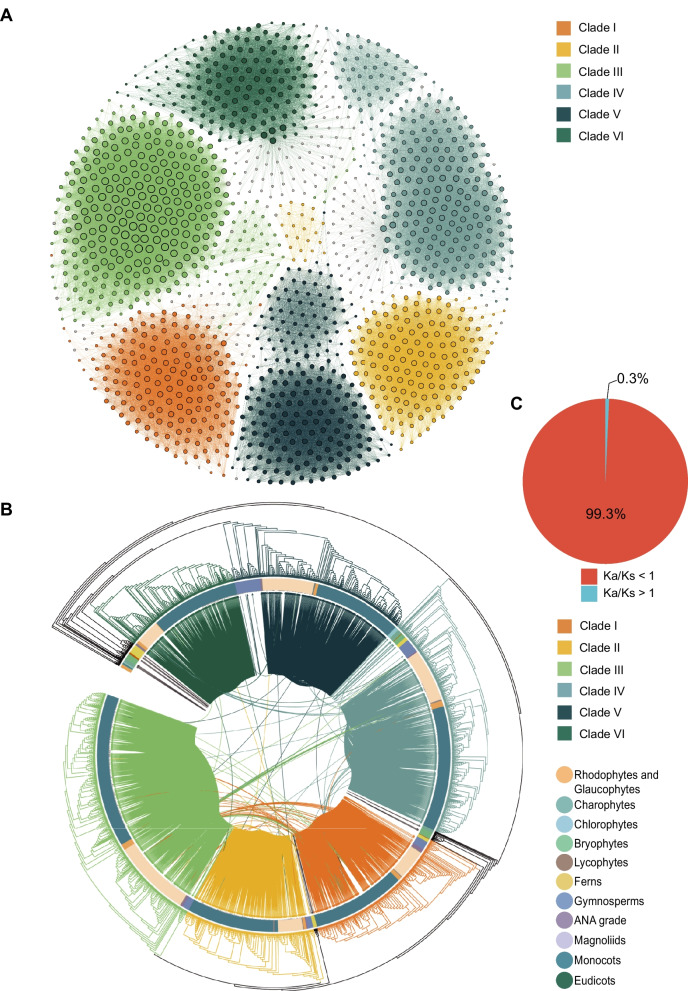
The collinearity network and collinearity relationships of LACS gene family members within and between different clades, as well as the proportion of Ka/Ks values in LACS gene family members. **A** The network clustering relationship of LACS gene family members based on the Clique percolation method with a K value of 4. The size of each node represents the number of connected edges. The network also clustered into six Clades (I, II, III, IV, V, a. VI), consistent with the phylogenetic tree in Fig. 2. **B** The collinearity relationship between species and within species of the LACS gene family in the phylogenetic tree. The line within the phylogenetic tree represents the collinearity of two LACS genes. **C** The pie chart represents the proportion of Ka/Ks values greater than or less than 1 for all LACS gene pairs

To further investigate the collinearity and evolutionary history of the LACS gene family, multiple alignment of whole-genome protein sequences within and between 166 species was performed. The synteny network contained a total of 6102 nodes (i.e., genes interconnected based on collinearity), which were connected by 65650 edges (i.e., pairwise collinearity between genes). Six main clusters were obtained by resolving and visualizing the LACS gene synteny network (Fig. 7A). These six clusters corresponded to the six clades (Clade I, Clade II, Clade III, Clade IV, Clade V, and Clade VI) from the phylogenetic analysis. Many collinear gene pairs were found via cluster analysis. The node size of Clade III in the clustering network was significantly larger than that of the other five clusters, indicating strong collinearity among the gene members in Clade III. The links between the collinearity relationships of all LACS genes and the phylogenetic trees shown in Fig. 2 were visualized using the ITOL website. The homologous relationships (homologous gene pairs) in the network clusters of all LACS genes corresponded to the relationships in the phylogenetic tree (Fig. 7B), indicating stronger collinearity between LACS family within the clades than among the clades.

We also calculated the ratios of nonsynonymous substitution rates (Ka) to synonymous substitution rates (Ks) (Fig. 7C, Supplementary Fig. S12, Table S13), and the results showed that homologous gene pairs with Ka/Ks values less than 1 accounted for 99.3% of the total gene pairs, while homologous gene pairs with Ka/Ks values greater than 1 accounted for 0.7%, indicating that the LACS gene family was subjected to strong purifying selection and strongly evolutionarily conserved.

## Discussion

LACS can activate free FAs to generate acyl-CoA thioesters, which are widely present in living organisms [7], including bacteria, mammals, algae and plants [51–54]. In this study, a total of 1785 LACS genes were identified in the genomes of 166 species widely distributed among plant lineages. There were significant differences in the number of LACS genes among species, and the average number overall showed an upward trend with the evolution of plant lineages. Phylogenetic analysis revealed six clades of LACS family genes (I, II, III, IV, V, and VI), which is consistent with the findings of previous studies [25]. The network clustering method has been applied to decipher evolutionary relationships in several gene families, such as the evolution of the SOT gene family in angiosperms, the ARF gene family in plants, and the lineage-specific evolution of MADS-box [55–57]. The result of network clustering analysis shows that there are six major clusters of 1785 LACS family genes, which is consistent with the result of phylogenetic tree. In the clustering network, all six clusters are primarily characterized by intra-cluster collinearity, with Clade III having the highest gene count and node size among the six clusters. The conserved gene collinearity within the phylogenetic tree also indicates that internal collinearity predominates among different clades.

Moreover, we performed motif detection on all LACS gene sequences using MEME and obtained 12 motifs. However, Ayaz et al. [25] obtained 20 motifs from 697 LACS gene sequences. This may be because the number of LACS gene sequences used in this study was 2.6 times greater than that used in previous studies, leading to the genes sharing fewer conserved motifs. In addition, motif pattern analysis revealed that among the six clades of plant LACS genes, the motif patterns of Clades I, II, and III were more similar and had a closer evolutionary relationship, while those of Clades V and VI were more similar and had a closer evolutionary relationship. Clade IV was located far from the other five clades. Previous studies have reported that acetyl-activating enzymes contain two highly conserved domains (AMP binding domain signature and ACS signature motif) [58, 59]. We found that these two conserved domains in Clade (I, II, III) and Clade (V, VI) were more similar (Fig. 5C), while those in Clade IV had lower similarity compared with those in plants on the other five clades. The similarity between the motif pattern and the conserved domains reflects the phylogenetic relationships between the six clades.

The LACS gene family belongs to a subfamily of acyl-activating enzymes (AAEs). Previous studies have shown that the evolution of the AAE superfamily in *Arabidopsis* involved multiple large-scale and small-scale genome duplication events [60]. Our results indicate that the evolution of the LACS gene family is contributed to by five types of duplication events, including WGD, TD, PD, TRD, and DSD, which is consistent with the findings of previous research [60]. Specifically, the proportions of DSD, TRD, and WGD are much greater than those of TD and PD, which are the main driving forces for the evolution of the LACS gene family in plants.

Genes were selected from six clades to construct a phylogenetic tree (Supplementary Figs. S1-S6). The results showed that the LACS genes identified in monocots in Clade III formed two clades (Supplementary Fig. S3). The LACS genes identified in eudicots by Clade IV formed two clades (Supplementary Fig. S4), each of which included the LACS family genes *AtLACS6* and *AtLACS7* from the model plant *Arabidopsis thaliana*. We speculate that the LACS family genes underwent duplication events in Clades III and IV of the phylogenetic tree, causing the expansion of the gene family and resulting in the number of LACS genes in Clades III and IV ranking first and second, respectively. This phenomenon has also been reported for the AGO, ALMT, and SLAC gene families [61, 62]. We used TrimAl software to adjust the multiple sequence alignment results from the parameter—gappyout to—automated1 for trimming and reconstructed the phylogenetic tree. Similar results were obtained (Supplementary Figs. S13-S18), indicating the high reliability of the results.

**Fig. 8:**
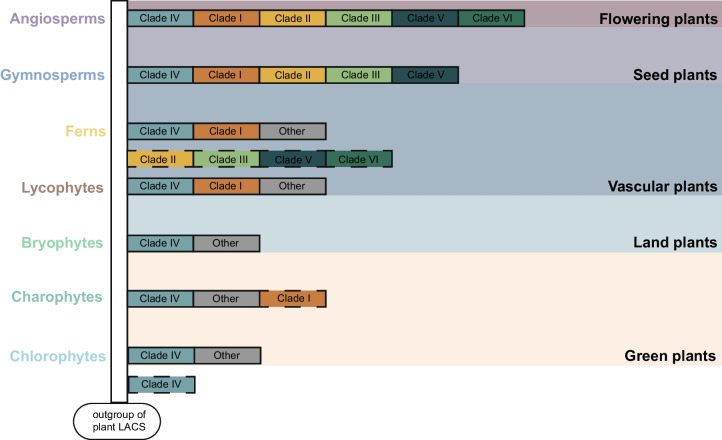
An evolutionary model of the LACS gene family in green plants. The solid-line box represents different clades, while the dotted-line box represents the ancestors of different clades. The LACS gene originated in green algae and was retained in green plants, with only one clade continuing to land plants. During the evolution of land plants toward vascular plants, the gene family expanded into two clades, followed by five clades in seed plants, and finally expanded into the current six clades in flowering plants

Previous studies have reported that the differentiation of plant LACS genes occurred before the origin of bryophytes [22], and Clades I-IV and V-VI differ from those in Chlorophyta and Rhodophyta, respectively [25]. In this study, we conducted in-depth research on each clade using more comprehensive sampling of plant lineages and selected appropriate species to reconstruct a phylogenetic tree based on the position of each clade in the plant evolutionary process to clearly describe the origin and evolutionary history of the LACS family in plants. Our results showed that the six clades of the LACS family appeared in the order Clade IV, Clade I, Clade (II, III, V), and Clade VI (Fig. 3B, C), and there were differences in the origin and possible ancestors of the different clades. Sixty-six LACS genes (named Other Class Genes) were independent of the six clades (Fig. 2). This finding is consistent with the findings of Ayaz et al. [25]. Clade (I, II, III, IV, V) shares a common ancestor with the corresponding LACS genes of the outgroup from Other Class Genes. We speculate that the ancestors of plant LACS genes differentiated into genes on different clades after a certain plant evolutionary event, while the Other Class Genes were not affected by this even and retained the ancestral characteristics. However, the Other Class Genes gradually disappeared after multiple plant evolutionary events. We constructed an evolutionary model for plant LACS genes (Fig. 8). The LACS gene in plants originated in chlorophytes, with the first clade of Clade IV appearing. After green plants originated, they evolved into vascular plants, and the second clade, Clade I, appeared. Subsequently, the vascular plants evolved into seed plants, and Clade (II, III, V) appeared. Finally, Clade VI emerged as the last clade along with the evolution of flowering plants. In addition, we speculated on the possible ancestors of different clades. The ancestors of Clade IV may be the ancestors of green plants, such as Rhodophyta. The ancestors of Clade I may have been the ancestors of land plants, and they may have originated in Charophyta. Clade (II, III, V, VI) appeared in seed plants, and its ancestors may have existed before ferns. The specific locations of these clade ancestors require further research. During this evolutionary process, the origin of Other Class Genes occurred before the appearance of all six clades, and their traces can be found in the ancestors of plants. The evolutionary relationships of these genes are relatively complex (Supplementary Fig. S9).

## Conclusions

This study identified 1785 LACS genes from the genomes of 166 plant species. The LACS family mainly consists of six clades (I, II, III, IV, V, and VI). The origins of the clades differ. Clade IV originated in chlorophytes and gave rise to the LACS gene in green plants. Clade I originated in vascular plants, Clade (II, III, V) originated in seed plants, and Clade VI originated in flowering plants. Among these six clades, Clade IV was relatively independent, while Clade (I, II, III) and Clade (V, VI) had more similar structures and closer evolutionary relationships. DSD and TRD are the main forces driving the evolution of plant LACS genes, leading to differences in evolutionary time and structural and functional diversity among different clades of the plant LACS family. The results of the collinearity expression network and collinear relationship inference show that each clade is mainly characterized by internal collinearity, and there are fewer cases of collinearity between clades. The Ka/Ks results also support this conclusion, with approximately 99.3% of homologous gene pairs having Ka/Ks < 1, indicating a strong influence of purifying selection. The results of this study provide new insights into the origin, evolutionary history, and phylogenetic relationships of different clades of plant LACS genes.

### Supplementary Information

Supplementary Material 1.

Supplementary Material 2.

## Data Availability

All data are available in the main text and supplemental materials.

## Abbreviations

LACS: Long-chain acyl-CoA synthetase
FAs: Fatty acids
LCFAs: Long-chain fatty acids
MCFAs: Medium-chain fatty acids
SCFAs: Short-chain fatty acids
ACS: Acyl-CoA synthetase
VLACS: Very long-chain acyl-CoA synthetase
MACS: Medium-chain acyl-CoA synthetase
SACS: Short-chain acyl-CoA synthetase
CoA: Coenzyme A
ATP: Adenosine Triphosphate
AMP: Adenosine Monophosphate
TAG: Triglyceride
Ka: Nonsynonymous
Ks: Synonymous
WGD: Whole-genome duplication
TD: Tandem duplication
PD: Proximal duplication
TRD: Transposed duplication
DSD: Dispersed duplication
AAEs: Acyl-activating enzymes
