# Supplementary material for: Evolutionary origin and gradual accumulation with plant evolution of the LACS family
Source: BMC Plant Biol. 2024 May 31;24:481. doi: 10.1186/s12870-024-05194-2 (PMC11140897; doi:10.1186/s12870-024-05194-2)
Supplement: Supplementary file 2 — Supplementary Material 2. [file 12870_2024_5194_MOESM2_ESM.pdf]

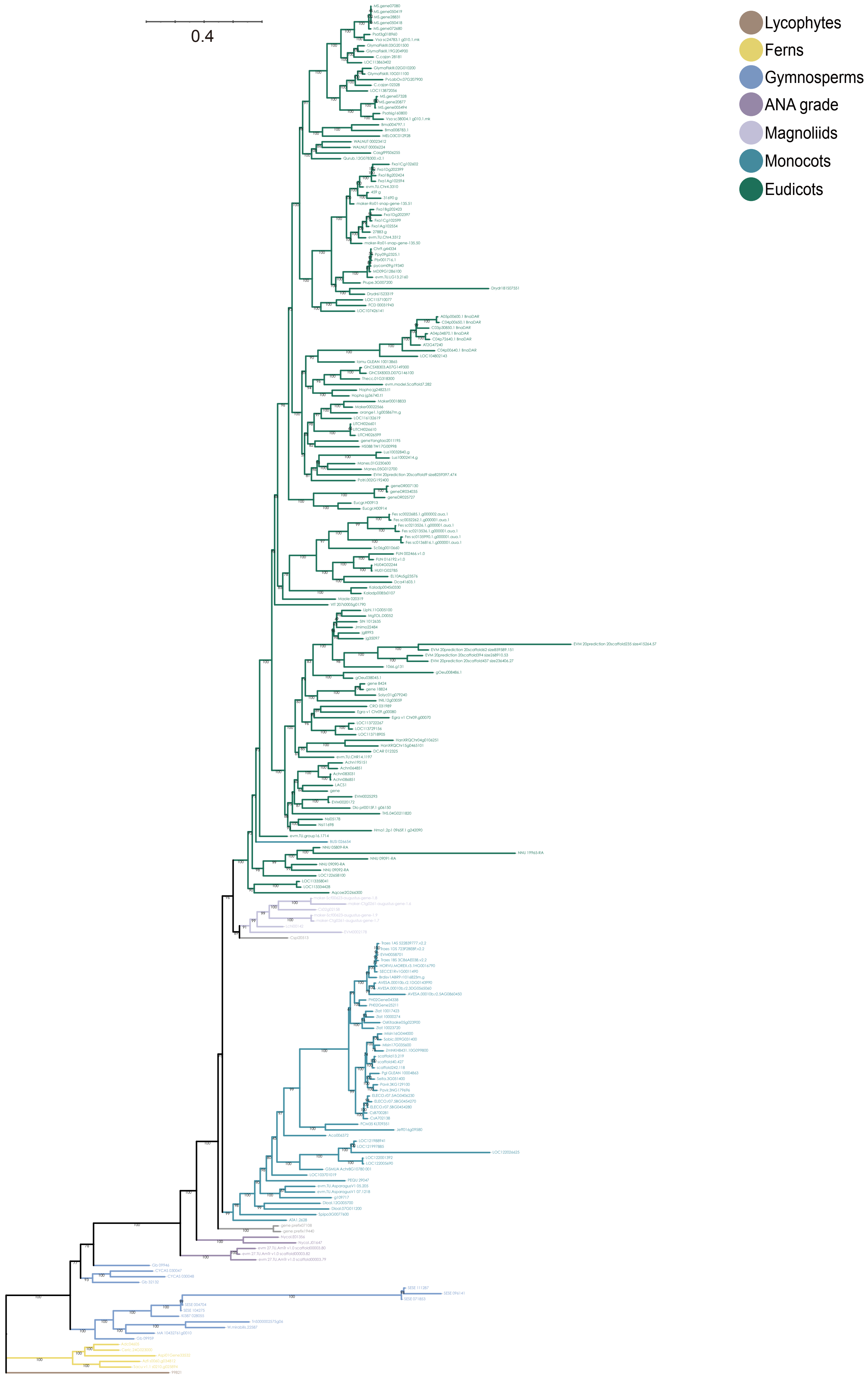

Fig. S1 The phylogenetic relationships of the LACS gene family from Clade I. The results of multiple sequence alignment were trimmed using TrimAl software with parameter -gappyout. The colors of different branches and labels represent different phyto-groups.

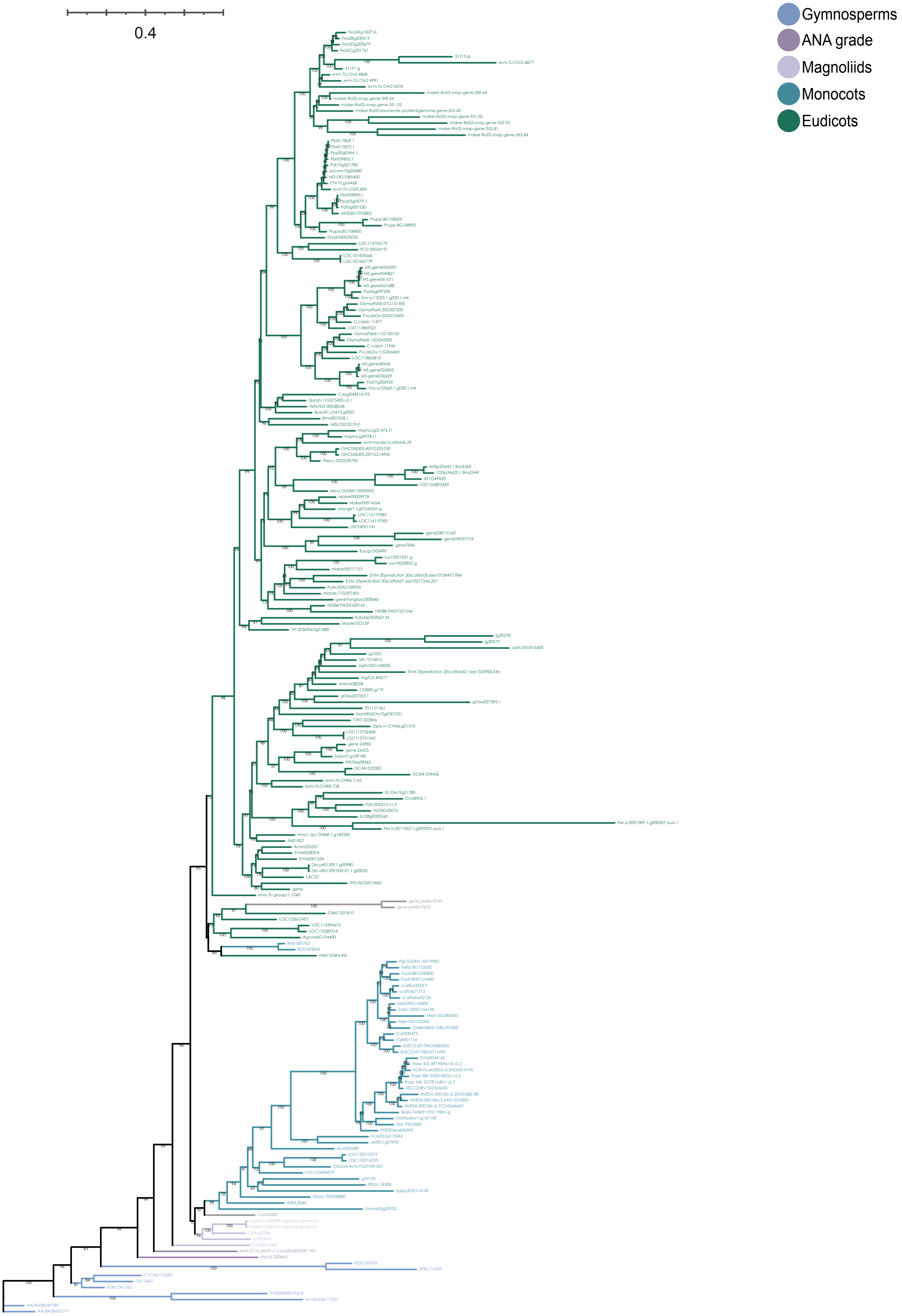

Fig. S2 The phylogenetic relationships of the LACS gene family from Clade II. The results of multiple sequence alignment were trimmed using TrimAl software with the parameter -gappyout. The colors of different branches and labels represent different phyto-groups.

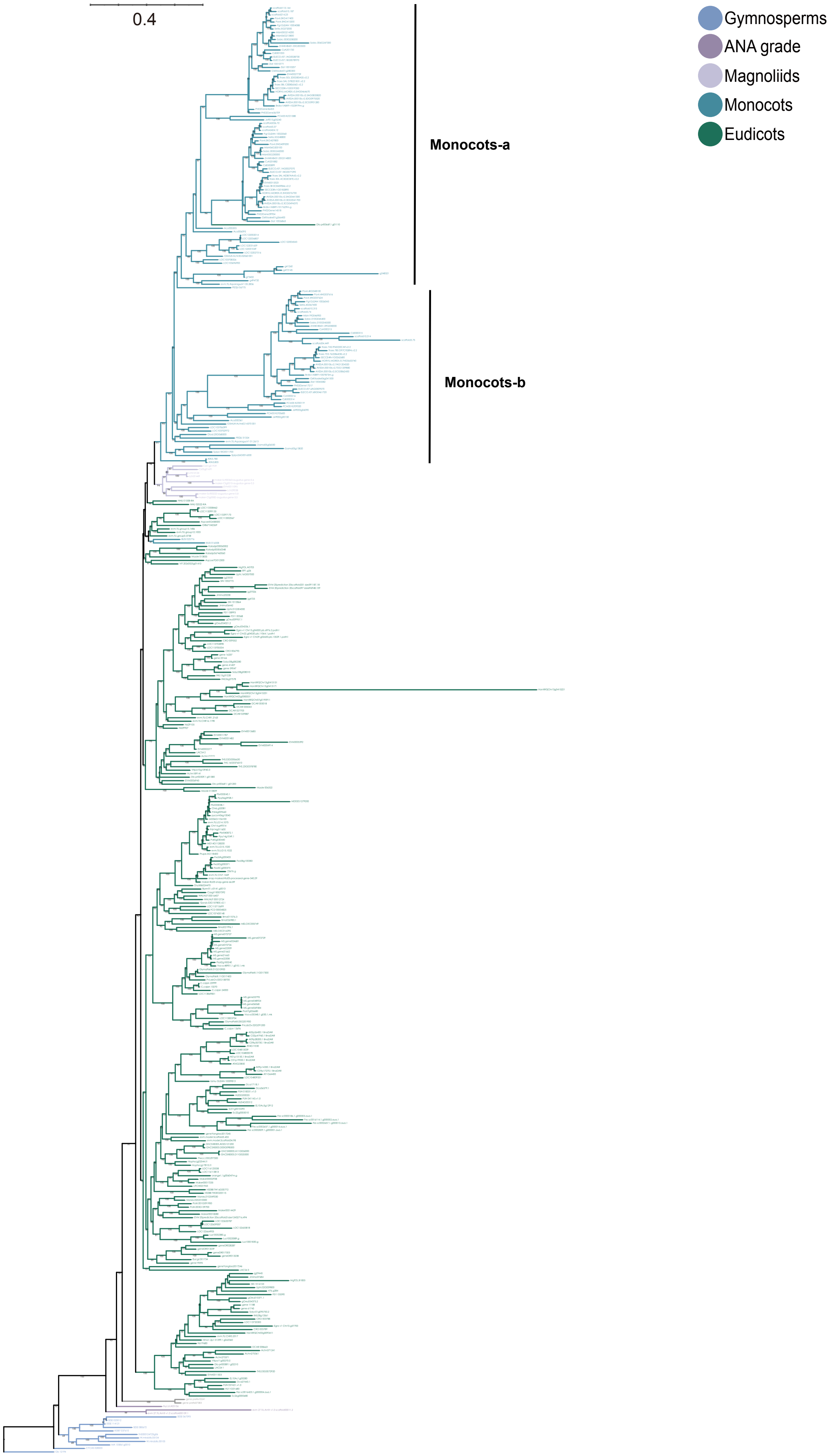

Fig. S3 The phylogenetic relationships of the LACS gene family from Clade III. The results of multiple sequence alignment were trimmed using TrimAl software with the parameter -gappyout. The colors of different branches and labels represent different phyto-groups.

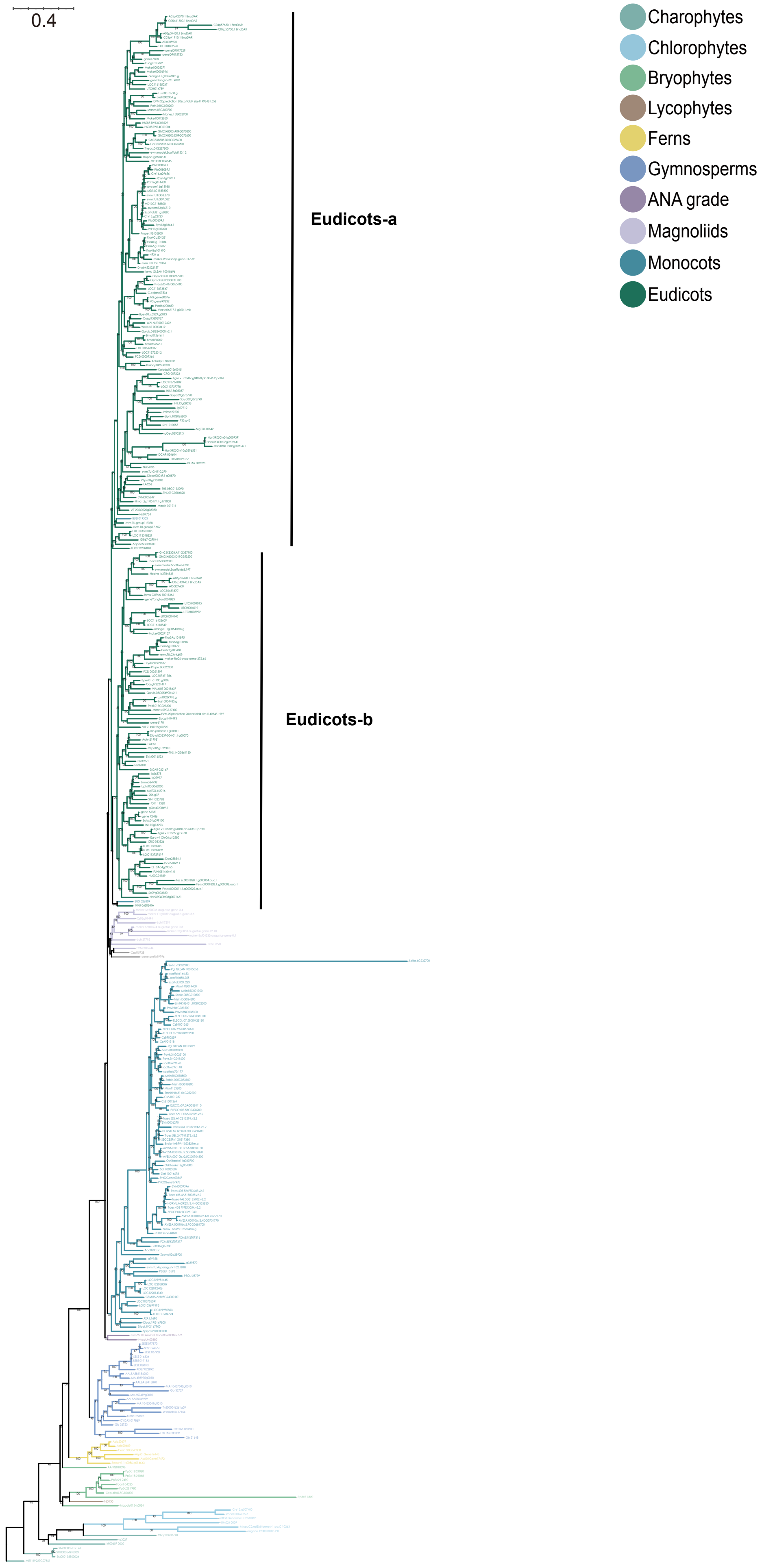

Fig. S4 The phylogenetic relationships of the LACS gene family from Clade IV. The results of multiple sequence alignment were trimmed using TrimAl software with the parameter -gappyout. The colors of different branches and labels represent different phyto-groups.

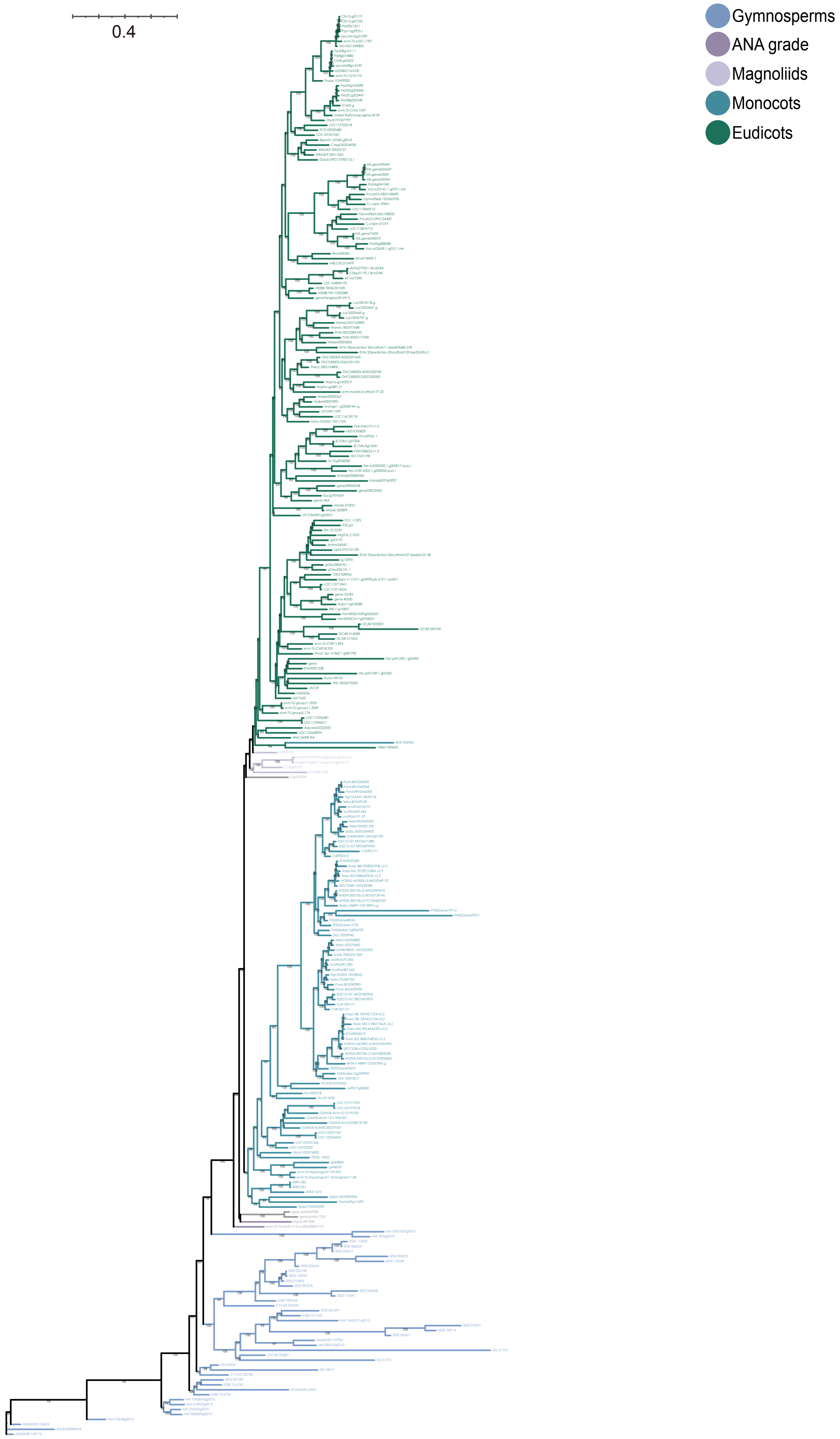

Fig. S5 The phylogenetic relationships of the LACS gene family from Clade V. The results of multiple sequence alignment were trimmed using TrimAl software with the parameter -gappyout. The colors of different branches and labels represent different phyto-groups.

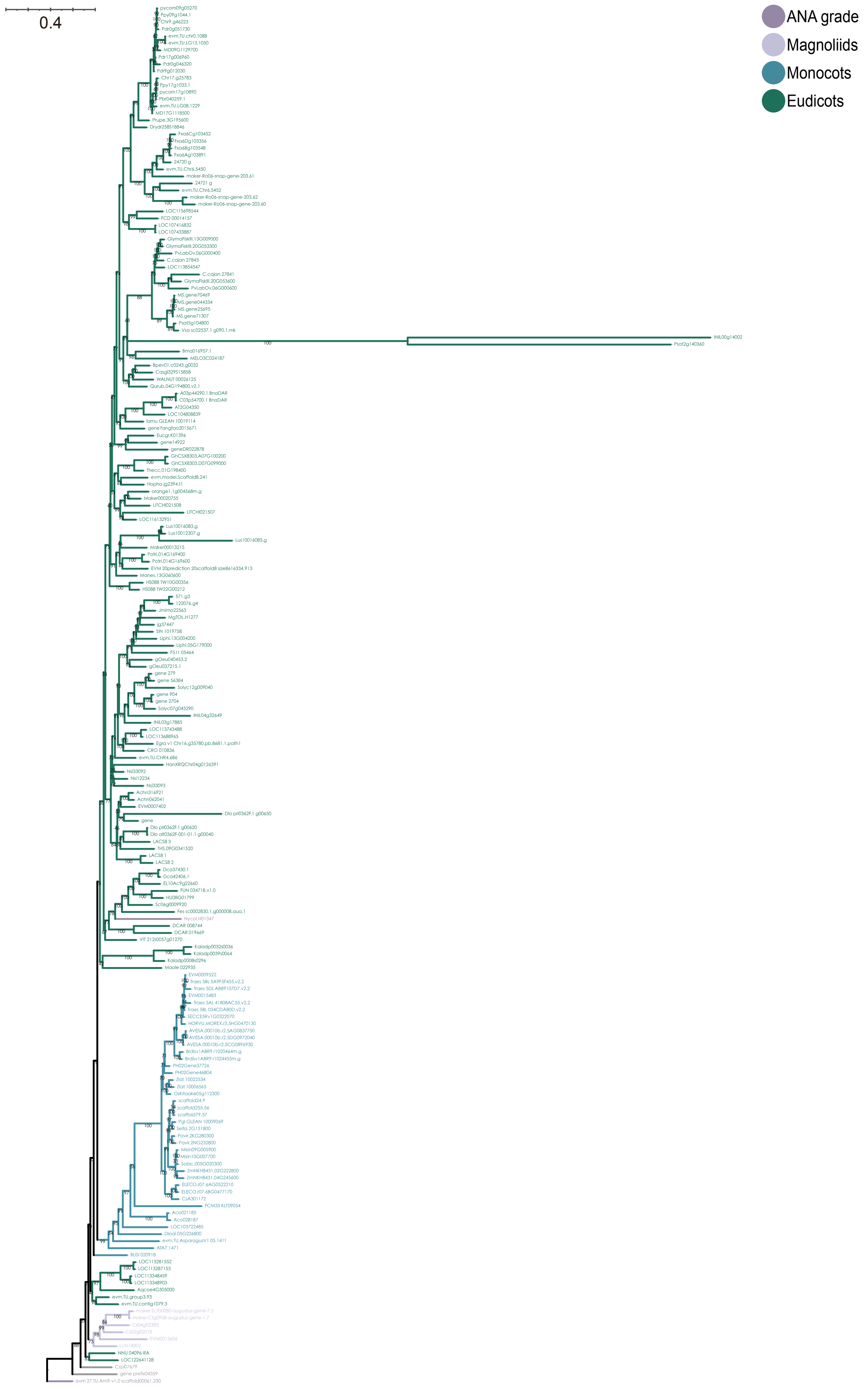

Fig. S6 The phylogenetic relationships of the LACS gene family from Clade VI. The results of multiple sequence alignment were trimmed using TrimAl software with the parameter -gappyout. The colors of different branches and labels represent different phyto-groups.

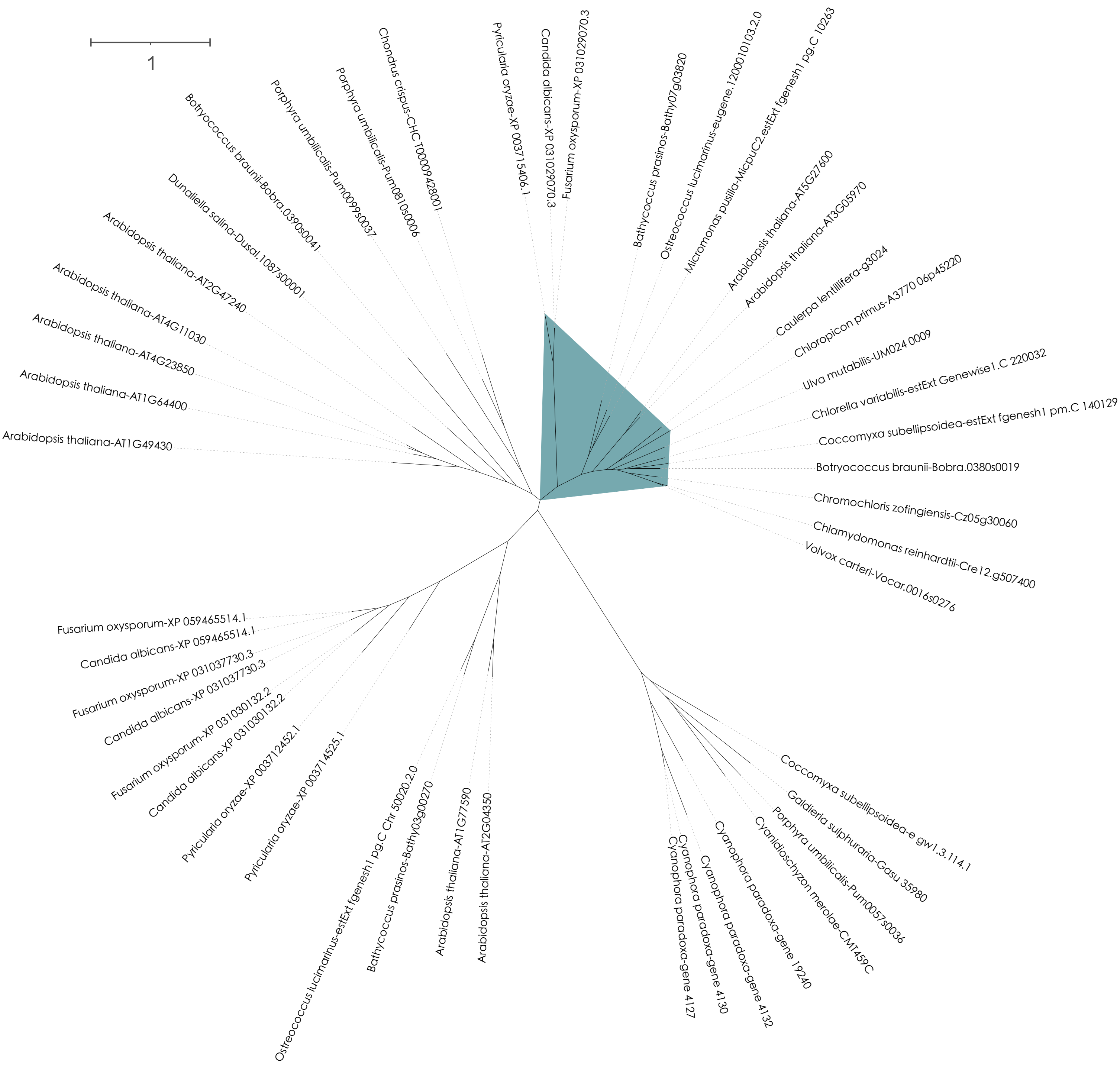

Fig. S7 The phylogenetic relationships between LACS genes in green plants and their outgroups. The branches marked with colors are related to the differentiation of the green plant Clade IV. The species used for the evolutionary tree are listed in Table S4.

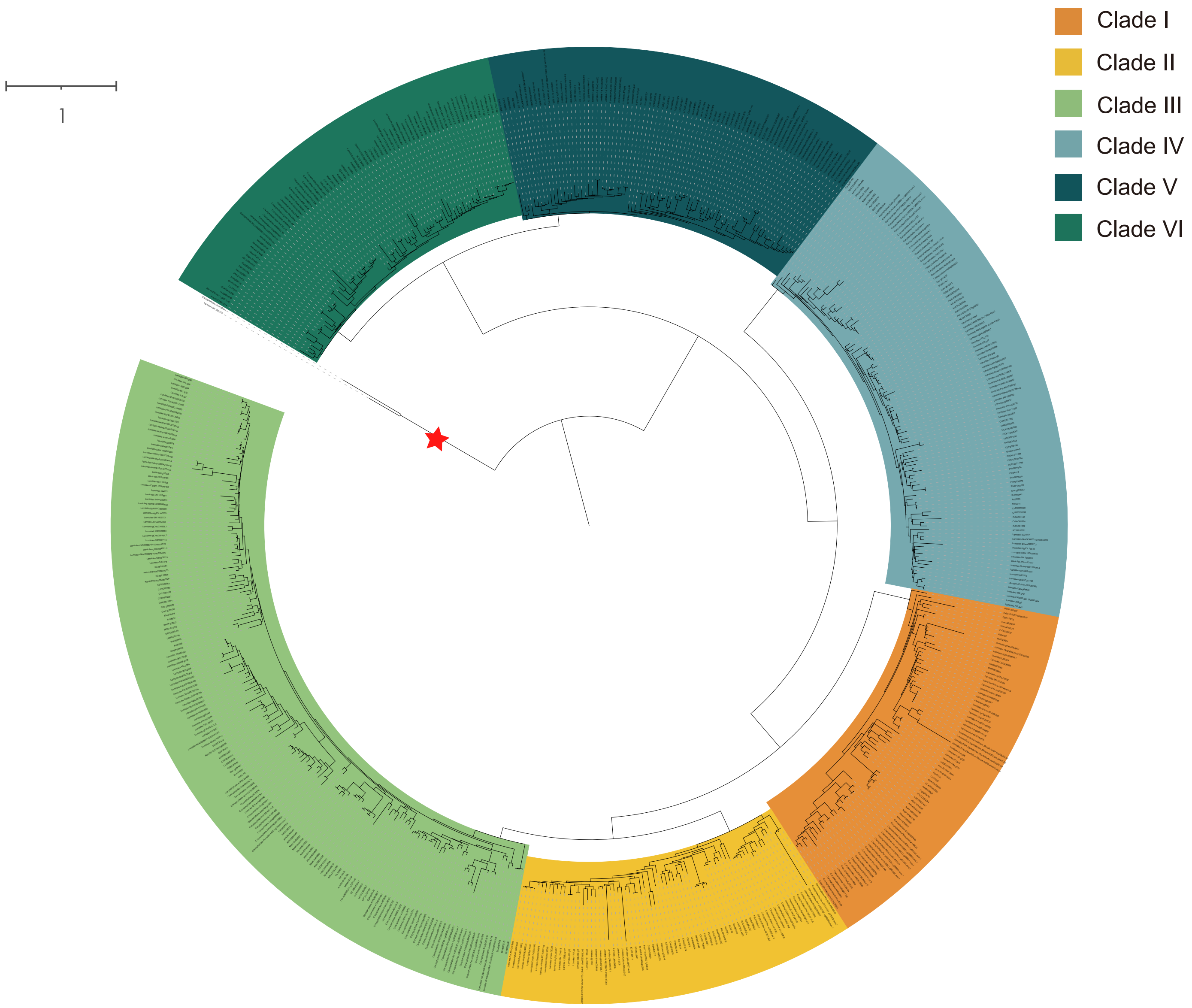

Fig. S8 Phylogenetic tree of LACS genes between the Caryophyllales and Lamiales orders. The red asterisk indicates the branching of two LACS genes of the Other Class Genes group from two dicotyledons identified among 166 species. The species used for the evolutionary tree are listed in Table S9.

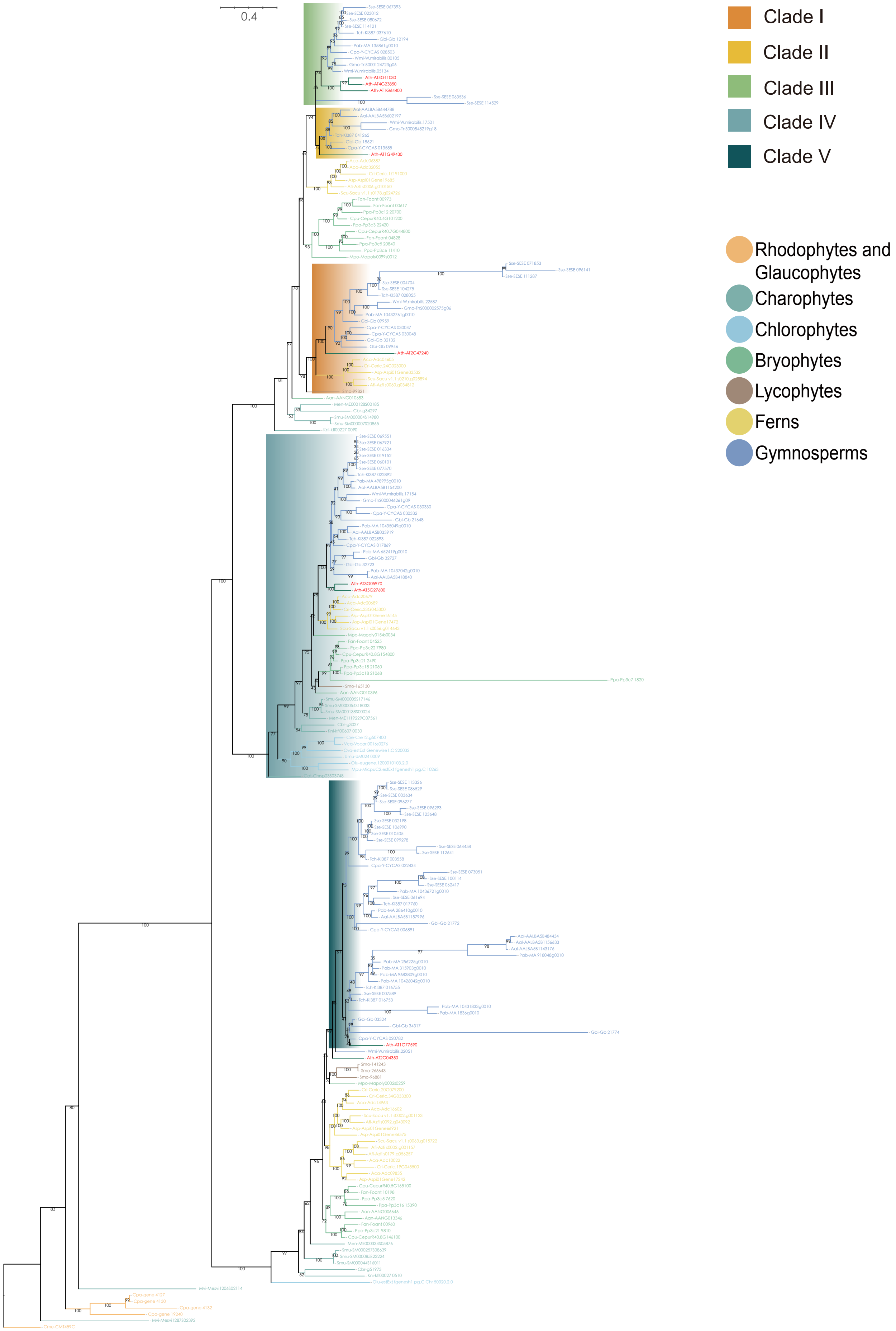

Fig. S9 The phylogenetic relationships of Other Class Genes. Different background colors represent different branches, and the colors of different branches and labels represent different phyto-groups. The species used for the evolutionary tree are listed in Table S10.

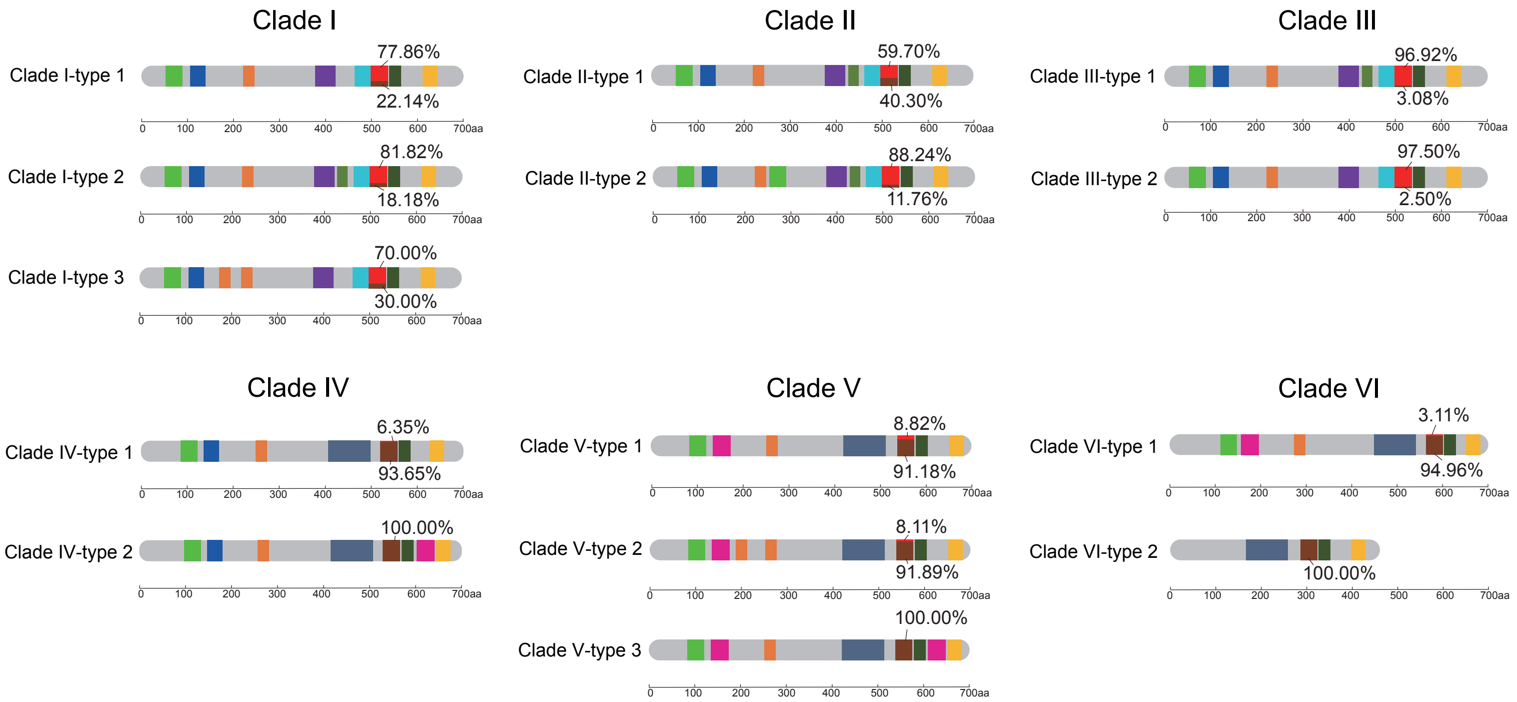

Fig. S10 Ration of motif2/3. We record motif 2 and motif 3 as the same type for each clade due to motif 2 and motif 3 appear at the same position in the gene sequence, and calculate their proportion in that type based on the number of occurrences of motif 2 and motif 3.

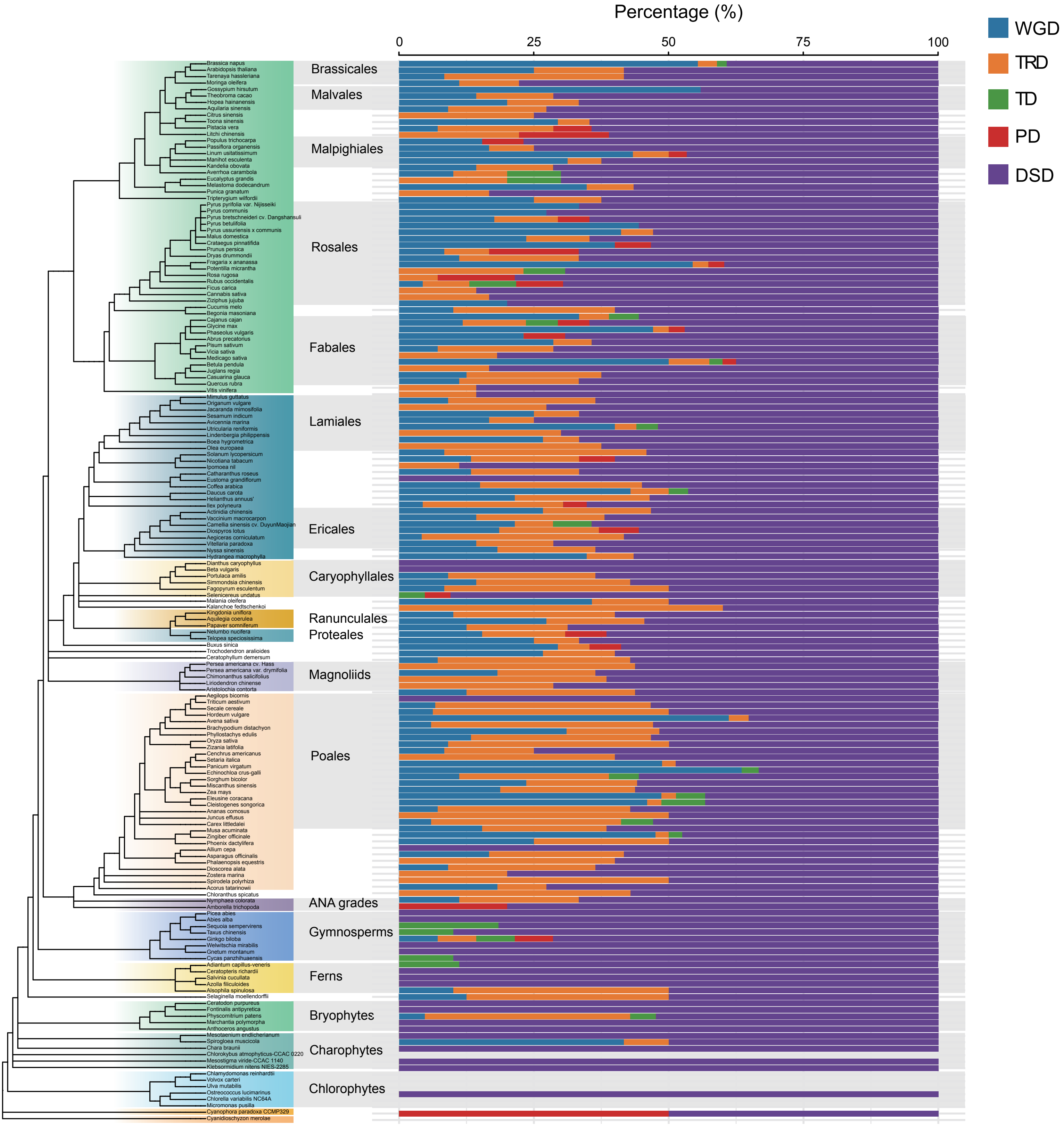

Fig. S11 The proportion of pairs of LACS gene family members from five duplication events (WGD, TD, PD, TRD and DSD) in 166 species genomes. The classification tree of 166 species was obtained from the NCBI Taxonomy Common Tree (https://www.ncbi.nlm.nih.gov/Taxonomy/CommonTree/www-cmt.cgi).

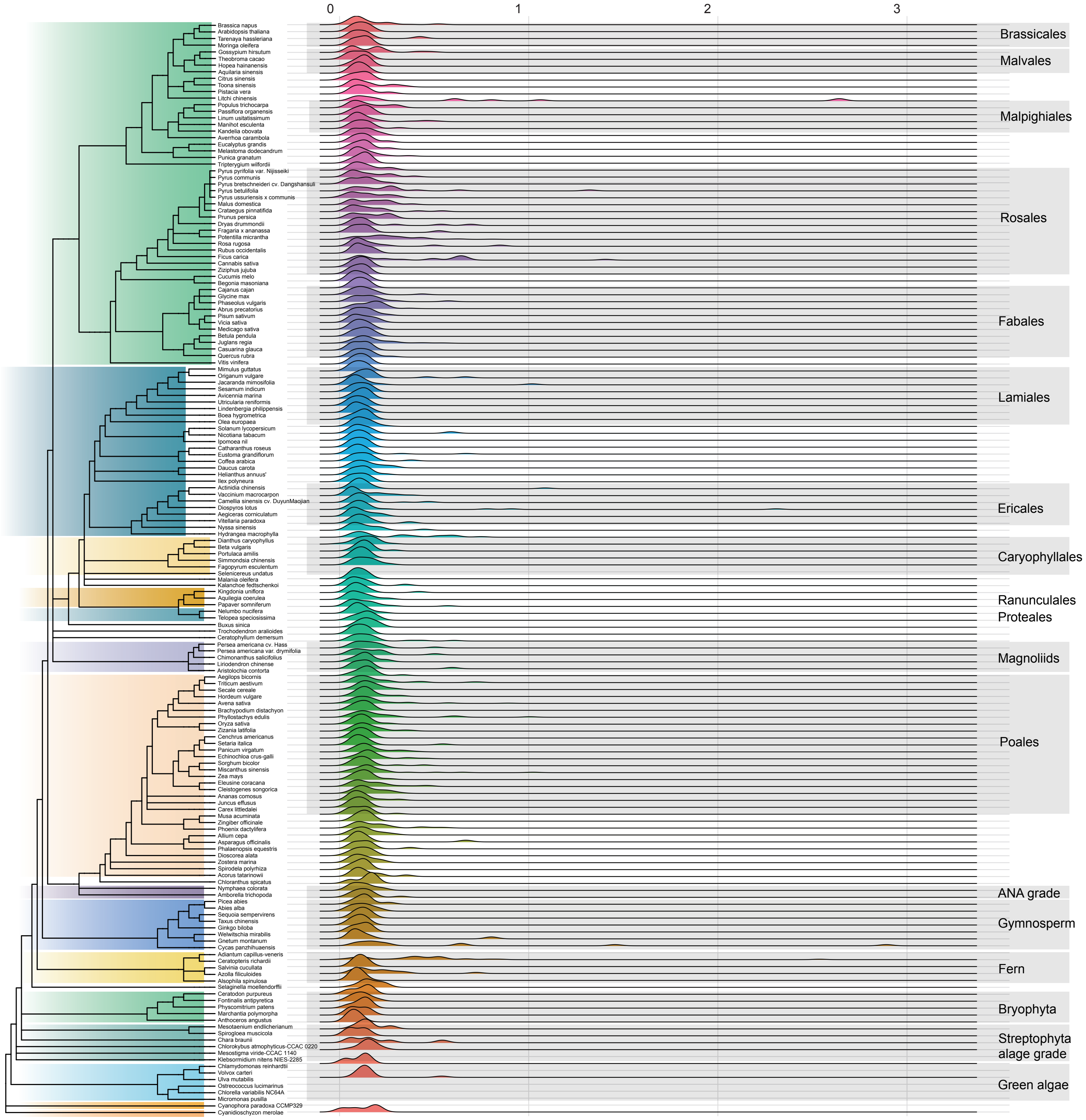

Fig. S12 Density plot of Ka/Ks values of LACS genes with different duplicated gene pairs for each species.

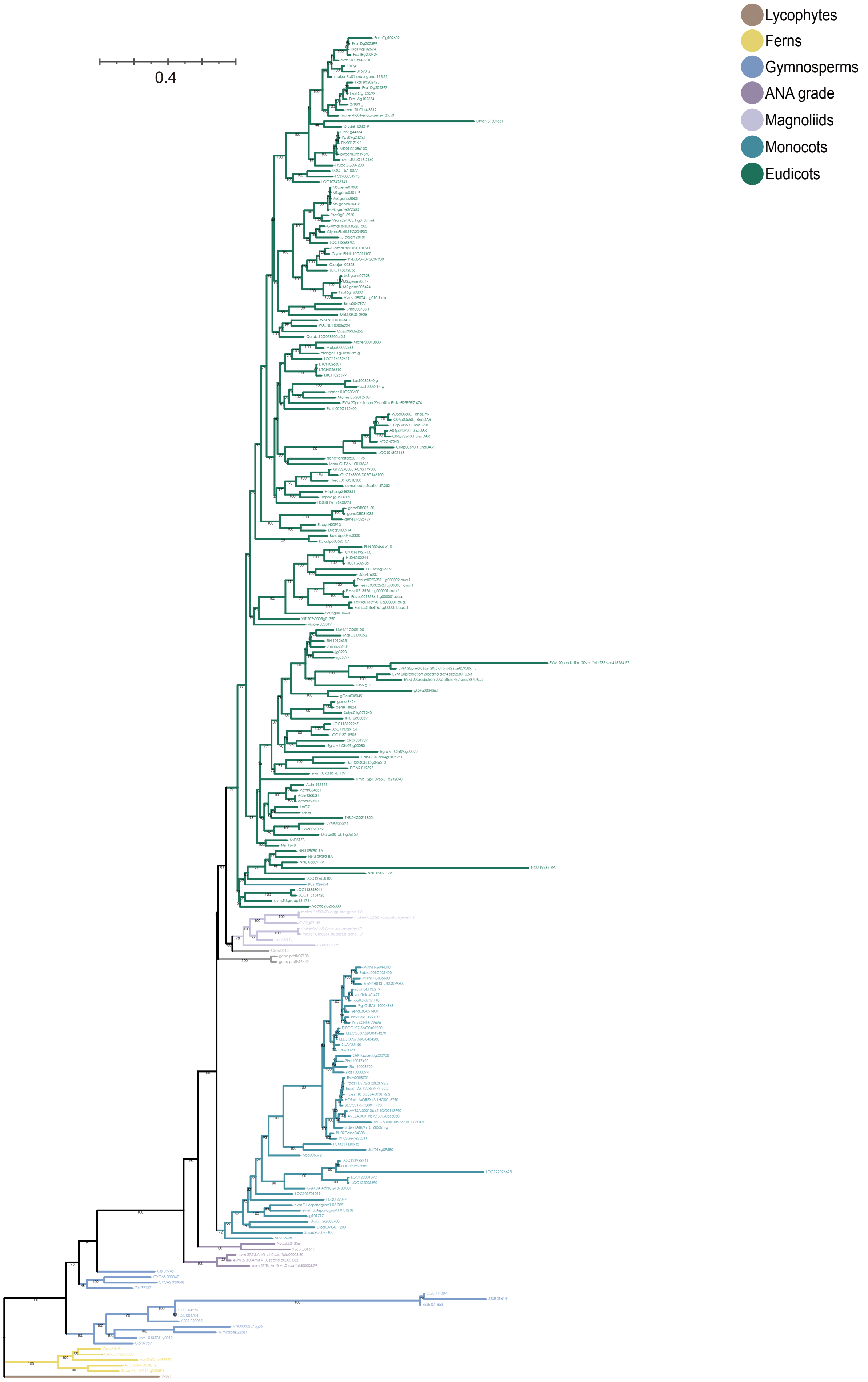

Fig. S13 The phylogenetic relationships of the LACS gene family from Clade I. The results of multiple sequence alignment were trimmed using TrimAl software with the parameter -automated1. The colors of different branches and labels represent different phyto-groups.

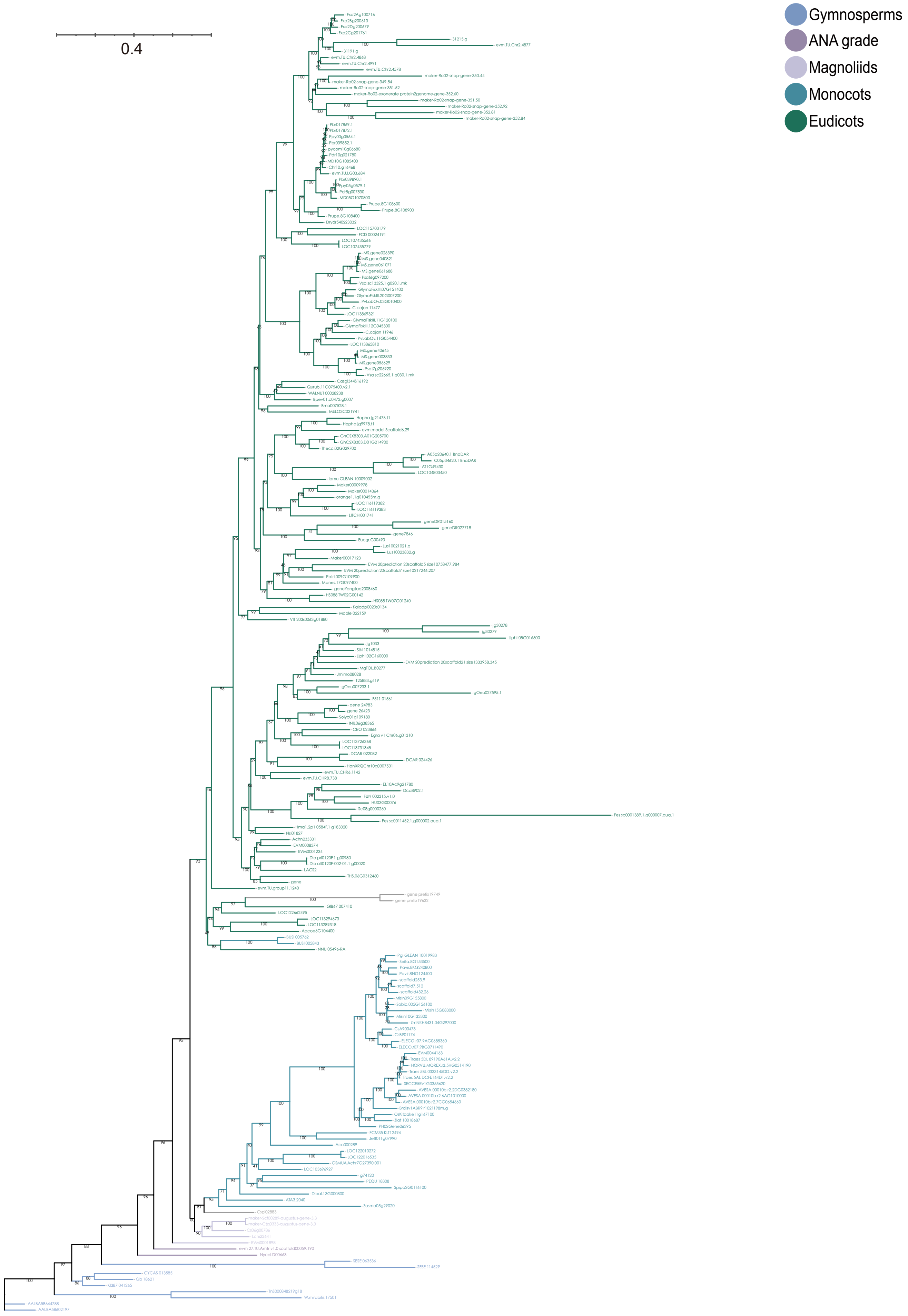

Fig. S14 The phylogenetic relationships of the LACS gene family from Clade II. The results of multiple sequence alignment were trimmed using TrimAl software with the parameter -automated1. The colors of different branches and labels represent different phyto-groups.

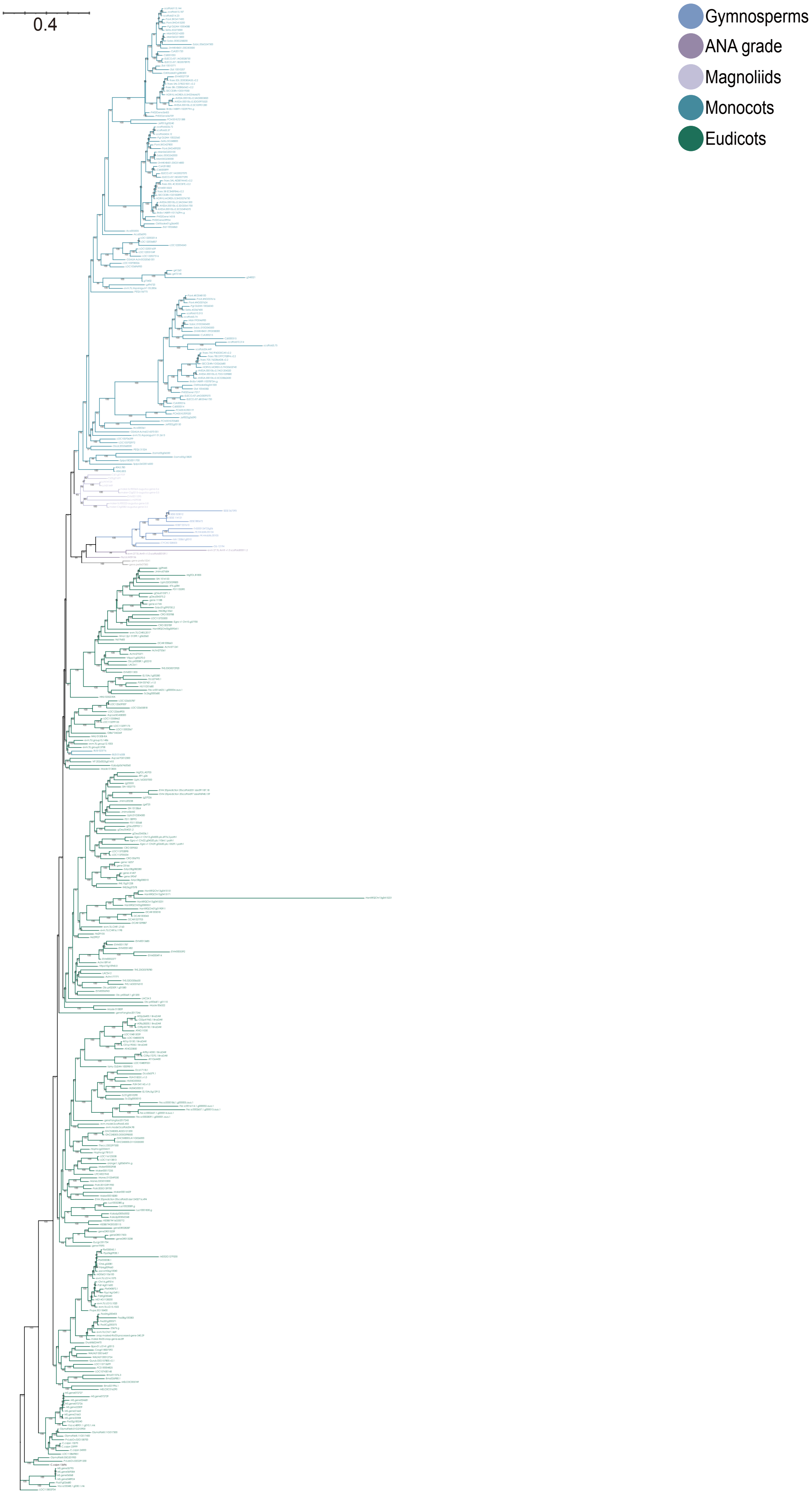

Fig. S15 The phylogenetic relationships of the LACS gene family from Clade III. The results of multiple sequence alignment were trimmed using TrimAl software with the parameter -automated1. The colors of different branches and labels represent different phyto-groups.

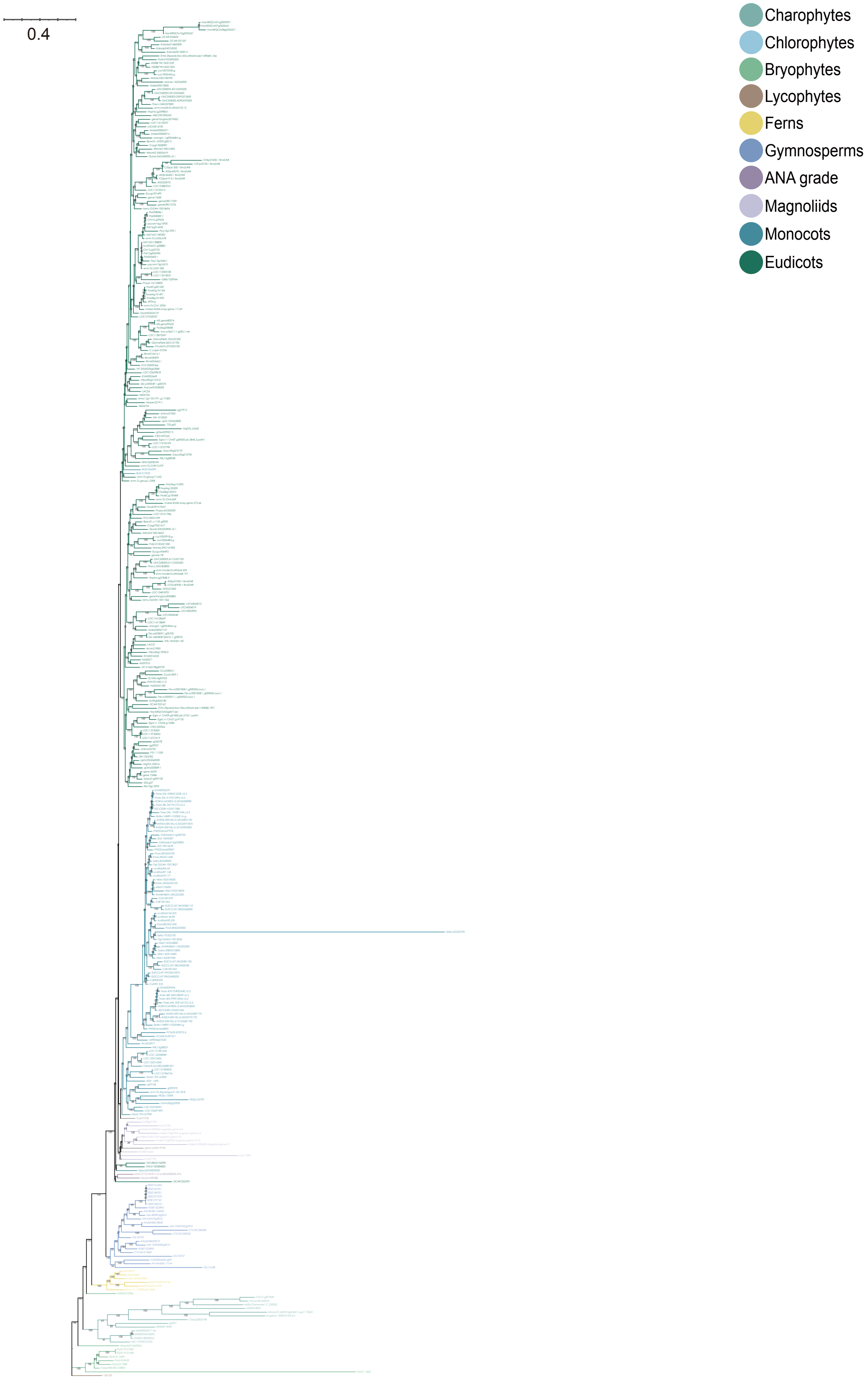

Fig. S16 The phylogenetic relationships of the LACS gene family from Clade IV. The results of multiple sequence alignment were trimmed using TrimAl software with the parameter -automated1. The colors of different branches and labels represent different phyto-groups.

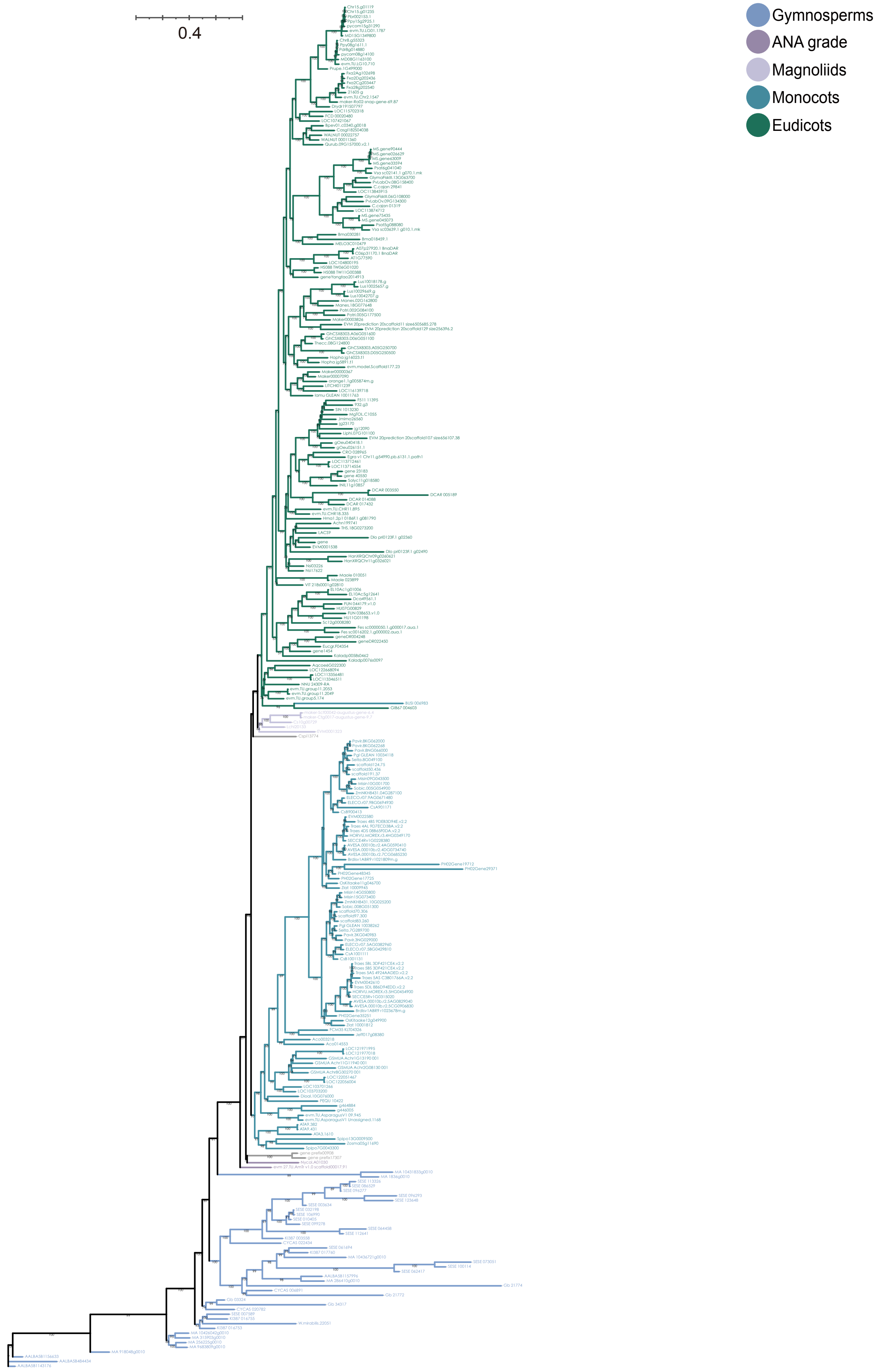

Fig. S17 The phylogenetic relationships of the LACS gene family from Clade V. The results of multiple sequence alignment were trimmed using TrimAl software with the parameter -automated1. The colors of different branches and labels represent different phyto-groups.

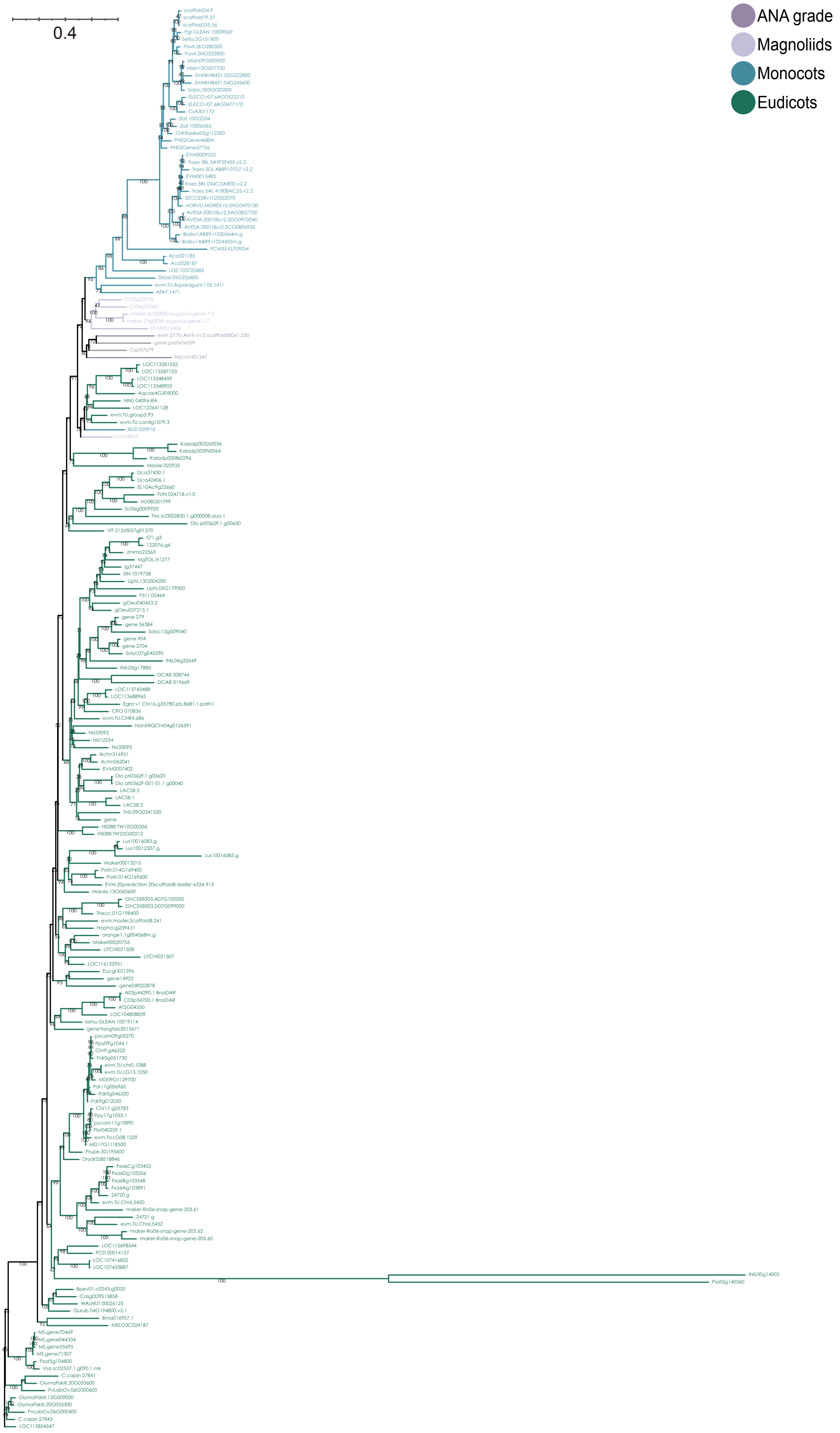

Fig. S18 The phylogenetic relationships of the LACS gene family from Clade VI. The results of multiple sequence alignment were trimmed using TrimAl software with the parameter -automated1. The colors of different branches and labels represent different phyto-groups.
